# Prediction of Drug Synergism between Peptides and Antineoplastic Drugs Paclitaxel, 5-Fluorouracil, and Doxorubicin Using In Silico Approaches

**DOI:** 10.3390/ijms24010069

**Published:** 2022-12-21

**Authors:** Nuno Vale, Mariana Pereira, Joana Santos, Catarina Moura, Lara Marques, Diana Duarte

**Affiliations:** 1 OncoPharma Research Group, Center for Health Technology and Services Research (CINTESIS), Rua Doutor Plácido da Costa, 4200-450 Porto, Portugal; 2 Department of Community Medicine, Health Information and Decision (MEDCIDS), Faculty of Medicine, University of Porto, Alameda Professor Hernâni Monteiro, 4200-319 Porto, Portugal; 3 CINTESIS@RISE, Faculty of Medicine, University of Porto, Alameda Professor Hernâni Monteiro, 4200-319 Porto, Portugal; 4 Institute of Biomedical Sciences Abel Salazar (ICBAS), University of Porto, Rua Jorge Viterbo Ferreira, 228, 4050-313 Porto, Portugal

**Keywords:** drug combination, cell-penetrating peptides, cancer therapy, in silico, drug synergism

## Abstract

Chemotherapy is the main treatment for most early-stage cancers; nevertheless, its efficacy is usually limited by drug resistance, toxicity, and tumor heterogeneity. Cell-penetrating peptides (CPPs) are small peptide sequences that can be used to increase the delivery rate of chemotherapeutic drugs to the tumor site, therefore contributing to overcoming these problems and enhancing the efficacy of chemotherapy. The drug combination is another promising strategy to overcome the aforementioned problems since the combined drugs can synergize through interconnected biological processes and target different pathways simultaneously. Here, we hypothesized that different peptides (P1–P4) could be used to enhance the delivery of chemotherapeutic agents into three different cancer cells (HT-29, MCF-7, and PC-3). In silico studies were performed to simulate the pharmacokinetic (PK) parameters of each peptide and antineoplastic agent to help predict synergistic interactions in vitro. These simulations predicted peptides P2–P4 to have higher bioavailability and lower T_max_, as well as the chemotherapeutic agent 5-fluorouracil (5-FU) to have enhanced permeability properties over other antineoplastic agents, with P3 having prominent accumulation in the colon. In vitro studies were then performed to evaluate the combination of each peptide with the chemotherapeutic agents as well as to assess the nature of drug interactions through the quantification of the Combination Index (CI). Our findings in MCF-7 and PC-3 cancer cells demonstrated that the combination of these peptides with paclitaxel (PTX) and doxorubicin (DOXO), respectively, is not advantageous over a single treatment with the chemotherapeutic agent. In the case of HT-29 colorectal cancer cells, the combination of P2–P4 with 5-FU resulted in synergistic cytotoxic effects, as predicted by the in silico simulations. Taken together, these findings demonstrate that these CPP6-conjugates can be used as adjuvant agents to increase the delivery of 5-FU into HT-29 colorectal cancer cells. Moreover, these results support the use of in silico approaches for the prediction of the interaction between drugs in combination therapy for cancer.

## 1. Introduction

Conventional chemotherapeutic drugs are widely used in cancer therapy; however, their success rate is commonly impaired by the development of adverse side effects and by the development of drug resistance, which together contribute to the inefficiency of chemotherapy, ultimately resulting in patient death [1]. The combination of conventional antineoplastic drugs with other molecules, such as cell-penetrating peptides, can be done to increase and target the delivery of the drug to the tumor site, therefore enhancing the bioavailability of the antineoplastic agent and improving the efficacy of the chemotherapy [2].

Cell-penetrating peptides (CPPs) are small peptide sequences that can be linked to other molecules to facilitate their cellular uptake, increase their blood circulation half-life, and help deliver the drugs into their sites of action more efficiently [3,4,5]. Sometimes, these peptides can display intrinsic anticancer activity and therefore may be used as anti-tumoral agents [6]. The exact mechanism by which CPPs enter cells has not yet been fully understood [7]. However, it is believed that some CPPs can directly penetrate via energy-independent pathways, such as by inverted micelle formation [8], pore formation [9], the carpet-like model [10], and the membrane thinning model [11], where the positively charged CPPs interact directly with the negatively charged components of the cellular membrane, such as heparin sulfate and the phospholipid bilayer, causing a transient destabilization of the membrane allowing the CPPs to penetrate cells [7]. CPPs can also enter cells by endocytosis, which can include phagocytosis for the uptake of large particles and pinocytosis for solute uptake [7]. This involves the inward folding of the outer surface of the plasma membrane, leading to the formation of vesicles that are internalized by cells [12]. Endocytosis of CPPs can also be mediated by receptors that cover the intracellular part of the membrane, like clathrin and caveolin, that help to form the vesicles after binding the extracellular molecule to the membrane receptor [13].

Previously, our research group has already evaluated different combination models involving the combination of antineoplastic drugs with repurposed drugs [14] and even using the combination of CPPs like CPP2 and CPP2-derivatives with antineoplastic and repurposed drugs [15] and have found that these compounds can successfully enhance the cytotoxic activity of chemotherapeutic agents in different cancer cell lines. In this way, based on these promising results, we hypothesized that (1) a novel cell-penetrating hexapeptide previously developed by our group (CPP6, Trp-Val-Pro-Thr-Leu-Lys(NH_2_)) and some derivates of this CPP could also help enhance the anticancer effects of different antineoplastic agents like paclitaxel (PTX), doxorubicin (DOXO), and 5-fluorouracil (5-FU) in different cancer cell lines, and (2) in silico approaches could be used to predict the drug synergism of these drug combinations, since, to our knowledge, this is the first time this type of simulation is being used to perform this kind of prediction.

CPP6 and several conjugates of this CPP were developed in a previous study of our research group to increase the uptake and selective release of methotrexate, a drug used for cancer treatment, in MCF-7 breast cancer cells [16]. CPP6 synthesis was inspired by two peptides (KLPVM and VPMLK) that were reported to have a high percentage of cell penetration in a family of CPP5 [17]. As CPP6 contains a considerably high ratio of positively charged amino acids, it acquires a cationic character that makes it able to target cancer cells that are characterized by an increased anionic nature of their membrane surface [18]. Therefore, we hypothesized that CPP6 could successfully increase and target the delivery of chemotherapeutic drugs to the tumor sites. We have found that the conjugation of CPP6 with the enzymatically cleavable linker GFLG (Gly-Phe-Leu-Gly) and short-length polyethylene glycol (PEG) polymer was able to (1) increase the blood circulation half-life, given by PEG, (2) enhance the ability to enter the cancer cells, mediated by CPP6, and (3) have a mechanism of drug release [16]. Moreover, it was found that CPP6 and its conjugates were able to successfully increase the target and delivery of methotrexate into breast cancer cells [16].

**Scheme 1:**
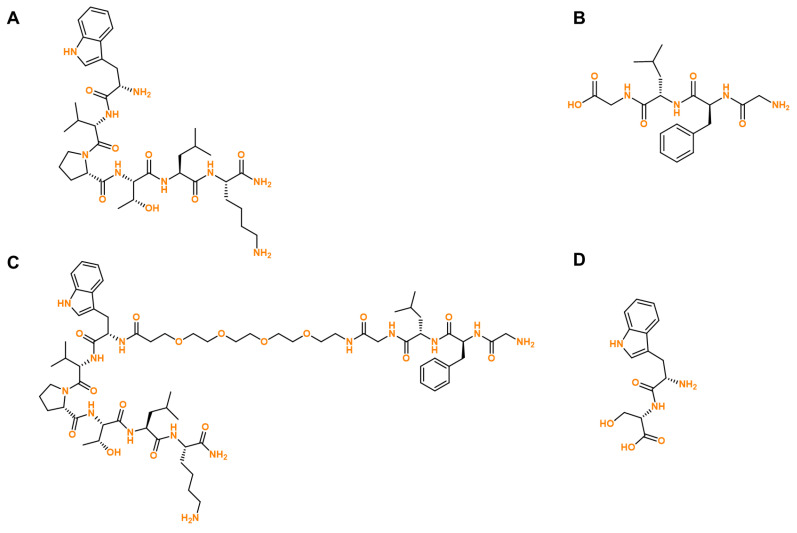
Chemical structure of the peptides evaluated in silico and in vitro in this manuscript. (**A**) P1, (**B**) P2, (**C**) P3, and (**D**) P4.

Therefore, here we evaluated if our previously synthesized peptides CPP6 (P1, Scheme 1A), CFLG (P2, Scheme 1B), and the conjugate CPP6-PEG-GFLG (P3, Scheme 1C) could be used in combination with conventional chemotherapy for breast, colorectal, and prostate cancer, aiming to find if these peptides could help to increase the permeability of cells to antineoplastic agents, increasing their bioavailability in tumor sites, and enhancing their anticancer efficacy. Structurally, P3 is a conjugate consisting of CPP6 (P1) and CFLG (P2) linked by PEG. The conjugation of CPP6 with PEG and GFLG theoretically stabilizes the intracellular pools of chemotherapeutic drugs in cancer cells [16]. PEG is the most applied polymer in drug delivery and remains the gold standard for stealth polymers. Taken together, this conjugation aimed to improve the pharmaceutical properties of CPP6, including better water solubility, lower immunogenicity, and prolonged blood circulation [16]. A dipeptide composed of the amino acids Trp and Ser (P4, Scheme 1D) was also included in this work as a comparator due to the ability of these amino acids to interact with the cellular membrane. Therefore, we coupled in silico studies with in vitro approaches to try to establish a relationship between the results obtained using computer-based software and in vitro results, trying to predict the interaction between drugs in combination. 

We first performed in silico studies to predict the permeability and other structure-related pharmacokinetic (PK) properties of each peptide and antineoplastic agent. Physiologically based pharmacokinetic (PBPK) models have proven useful for integrating all the parameters that influence the parameters related to the properties of the drugs and the physiological parameters specific to the species [19]. The modeling platform GastroPlus^®^, a mechanistically based simulation software, can be useful to predict these pharmacokinetic proprieties using oral advanced compartmental and transit (ACAT). The generic GastroPlus^®^ ACAT model provided reasonable predictions especially for biopharmaceutical classification system (BCS) class 1 compounds [20]. In addition, the ability of PBPK models to predict oral PK will also improve, providing a better tool for the discovery and development of new medicines [21,22,23,24], as drug combinations or drug synergism. 

Next, peptides were evaluated in vitro, both alone and combined with different chemotherapeutic drugs, in three cancer cell lines: MCF-7 (breast), HT-29 (colon), and PC-3 (prostate), to determine if these combinations could be used to enhance the anticancer potential of the antineoplastic drugs. The antineoplastic drugs such as PTX, 5-FU, and DOXO were selected to be used as reference chemotherapeutic drugs for breast, colon, and prostate cancer cells, respectively. Finally, after determining the most promising drug combinations, the interaction between each peptide and chemotherapeutic drug in the combination was evaluated to determine eventual synergistic interactions through the calculation of the combination index using the Chou-Talalay method [25]. 

Our in silico findings have predicted the peptides P2–P4 to have higher bioavailability and lower T_max_, as well as the chemotherapeutic agent 5-FU to have enhanced permeability properties over other antineoplastic agents, with P3 having a prominent accumulation in the colon. Therefore, we hypothesized that the combination of 5-FU with these peptides in vitro would lead to increased delivery of 5-FU into colorectal cancer cells, increasing the anticancer activity of this drug and possibly resulting in drug synergism between these two molecules. Indeed, our findings in MCF-7 and PC-3 cancer cells demonstrated that the combination of PTX and DOXO, respectively, with these peptides is not advantageous over a single treatment with the chemotherapeutic agent. Nevertheless, in the case of HT-29 colorectal cancer cells, the combination of P2, P3, and P4 with 5-FU resulted in synergistic cytotoxic effects, as predicted by the in silico simulations. 

Taken together, these results demonstrate that the combination of these peptides with 5-FU may enhance the delivery of the antineoplastic agent into colorectal cancer cells. Moreover, these findings support the idea that in silico simulations may be an important tool for the prediction of the interaction between drugs in combination interaction for cancer therapy.

## 2. Results and Discussion

### 2.1. In Silico Evaluation of the PK Properties of 5-FU, DOXO, and PTX

We first simulated the plasma concentration for 24 h of the chemotherapeutic agents 5-FU, DOXO, and PTX as well as the compartmental absorption of these drugs and other PK parameters using the GastroPlus^®^ software. These simulations were performed for an American individual, 30 years old, with an initial dose of 50 mg for 24 h. The simulations regarding the chemotherapeutic agents and peptides were run separately because these compounds were also administered separately to the cancer cells. 

**Figure 1:**
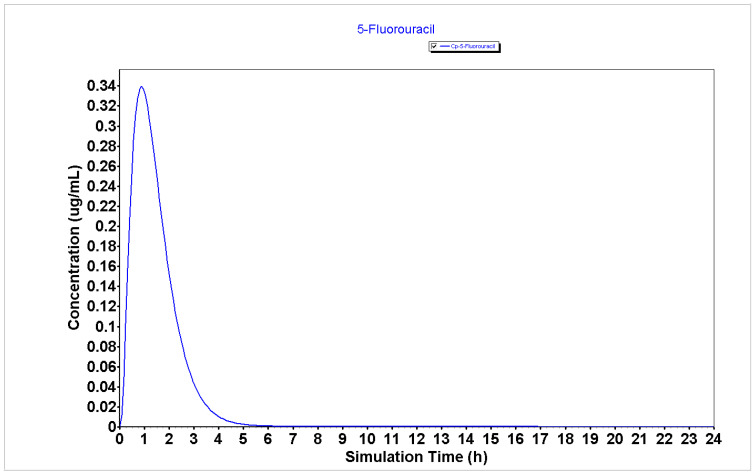
Plasma concentration of 5-FU generated using the GastroPlus^®^ software.

**Figure 2:**
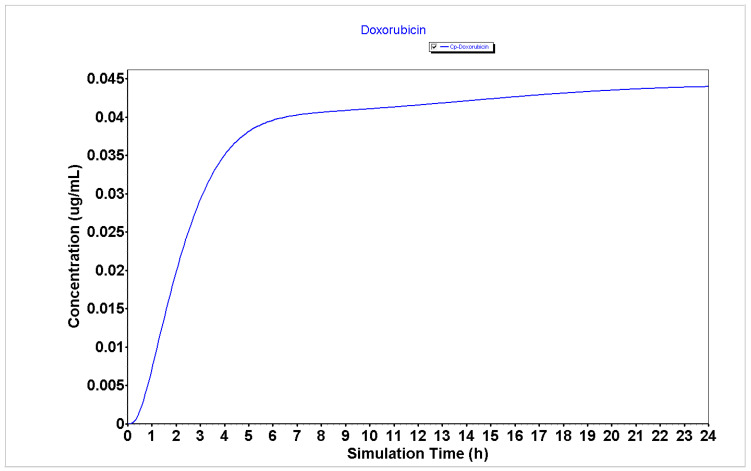
Plasma concentration of DOXO generated using the GastroPlus^®^ software.

**Figure 3:**
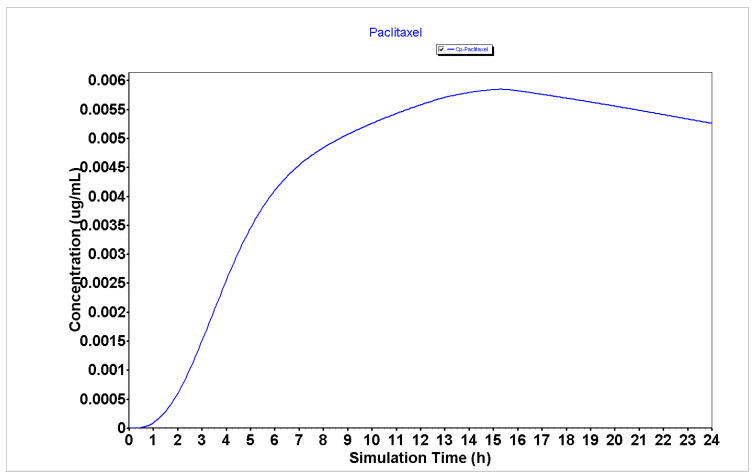
Plasma concentration of PTX generated using the GastroPlus^®^ software.

The detailed PK parameters obtained for 5-FU, DOXO, and PTX are described in Table 1 and the plasma concentration and compartmental absorption of each drug are represented in Figure 1, Figure 2 and Figure 3.

The results from Table 1 regarding the PK parameters for the GastroPlus^®^ simulation of 5-FU, DOXO, and PTX demonstrate that 5-FU has a higher P_eff_ (2.81 cm/s × 10^4^) than DOXO and PTX (0.26 and 0.21 cm/s × 10^4^, respectively), which may indicate that this drug will have enhanced ability to enter cancer cells and to exert cytotoxic effect than DOXO and PTX. Moreover, the analysis of the bioavailability (F) of 5-FU demonstrates it has a higher bioavailability than DOXO and PTX (55.66 vs. 49.40 and 5.47%, respectively), which may indicate this drug will be more available to interact with other molecules such as peptides than DOXO and PTX.

Analyzing the plasma concentration representations (Figure 1, Figure 2 and Figure 3), it is possible to verify that 5-FU (Figure 1) has an improved PK profile compared to DOXO and PTX (Figure 2 and Figure 3, respectively), characterized by a rapid absorption as well as a rapid elimination rate. Rapid elimination is especially important for chemotherapeutic drugs to avoid a prolonged accumulation of the drugs inside the organism that could further lead to the development of undesired side effects.

The compartmental absorption of 5-FU, DOXO, and PTX is represented in Figures S1–S3. These results demonstrate that 5-FU and DOXO have higher absorption in jejunum (Figures S1 and S2, respectively), while PTX accumulates most in the ascending colon (Figure S3).

Taken together, these results demonstrate that 5-FU may have an improved PK profile over DOXO and PTX, which may indicate better anticancer effects in cancer cells.

### 2.2. In Silico Evaluation of the PK Properties of Peptides P1–P4

**Figure 4:**
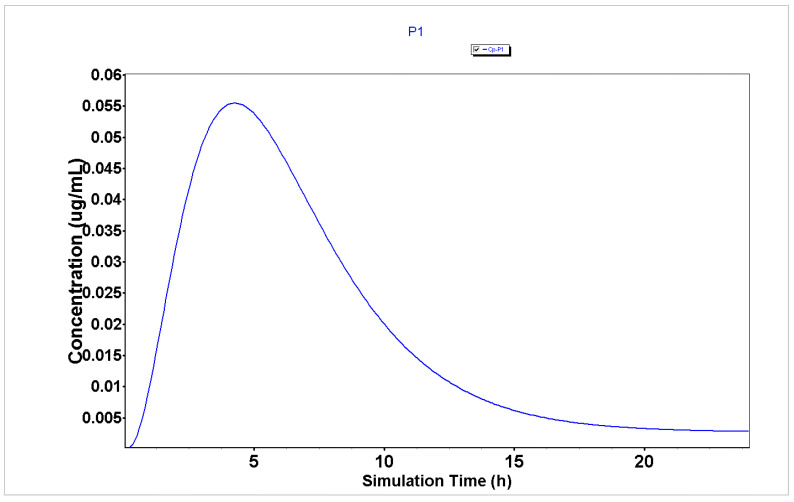
Plasma concentration of P1 generated using the GastroPlus^®^ software.

**Figure 5:**
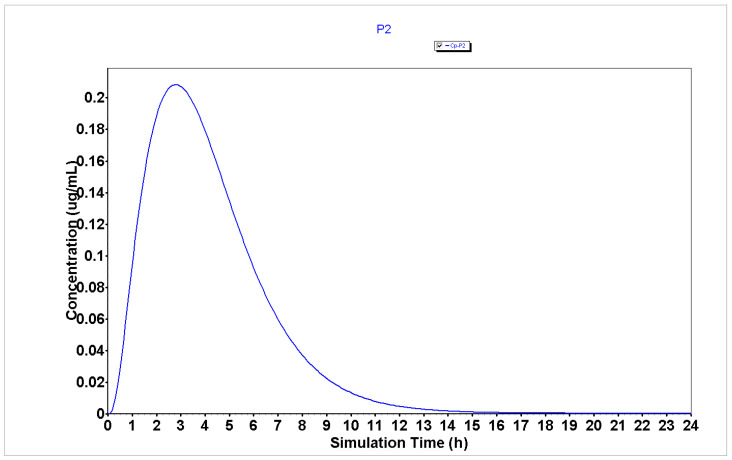
Plasma concentration of P2 generated using the GastroPlus^®^ software.

**Figure 6:**
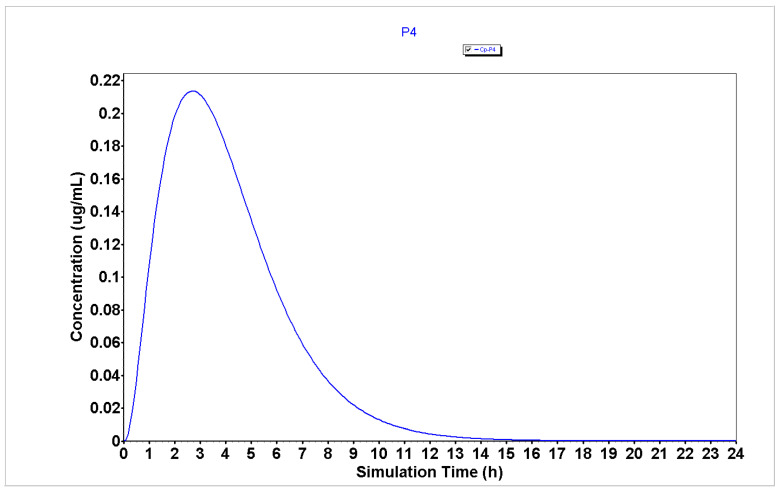
Plasma concentration of P4 generated using the GastroPlus^®^ software.

**Figure 7:**
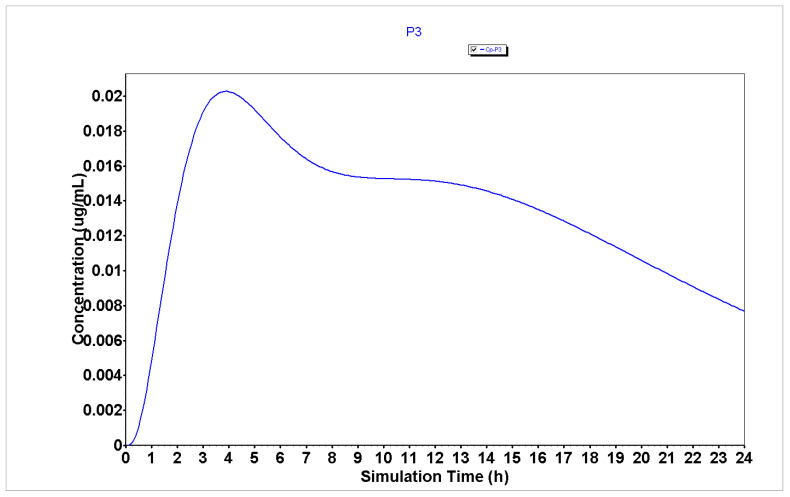
Plasma concentration of P3 generated using the GastroPlus^®^ software.

Then, we simulated the plasma concentration for 24 h of peptides P1–P4 as well as their compartmental absorption and other PK parameters using the GastroPlus^®^ software. These simulations were performed using the same parameters as previously described for 5-FU, DOXO, and PTX. These results are shown in Figure 4, Figure 5, Figure 6 and Figure 7 and Table 2.

The analysis of plasma concentration over time demonstrates that peptides P1, P2, and P4 (Figure 4, Figure 5 and Figure 6, respectively) are characterized by having a rapid absorption rate as well as an elimination profile. P3 (Figure 7) has a slightly different PK profile, being rapidly absorbed but having a reduced elimination rate compared to peptides P1, P2, and P4. A slower elimination rate can be specifically helpful when using peptides for enhancing drug transport to increase their time inside the organism. Some studies have suggested that some CPPs have enhanced uptake efficacy and delivery efficiency than other similar treatments, such as nanoparticles, while presenting less cytotoxicity [26]. Indeed, several peptides have already entered Phase I, Phase II and even, Phase III clinical trials [2]. Therefore, assuming these peptides also have an acceptable biosafety profile, their increased time inside the organism may not result in side effects. 

The analysis of PK parameters from Table 2 demonstrates that P2–P4 are characterized by having enhanced bioavailability (32.77, 17.73, and 37.45%, respectively) than P1 (13.15%), which indicates that these peptides will have more availability to interact with the chemotherapeutic agents when administered in combination than P1. Moreover, peptides P2–P4 are characterized by having lower T_max_ (2.8, 3.84, and 2.72 h, respectively) than peptide P1 (4.24 h), which indicates that these peptides will also interact faster and produce a rapid therapeutic effect when combined with the chemotherapeutic agents than P1.

**Figure 8:**
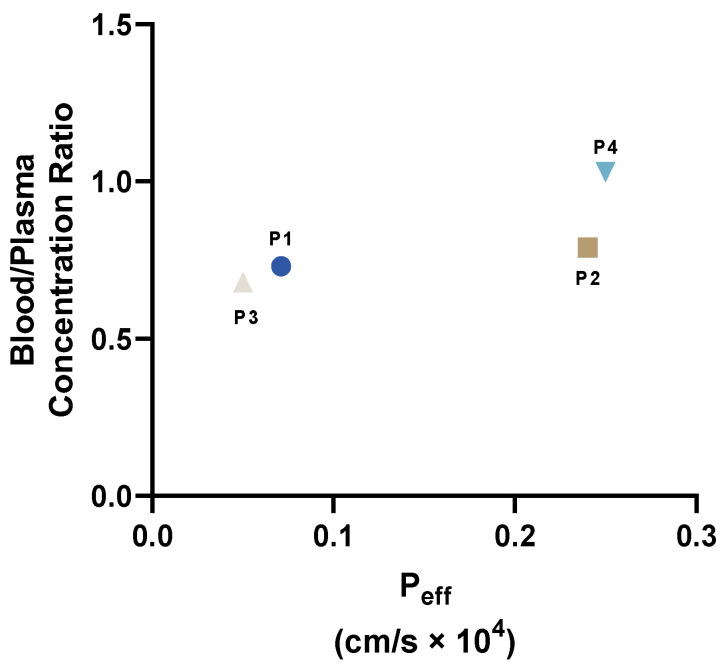
Representation of Blood/Plasma concentration ratio versus P_eff_ for peptides P1–P4.

The representation of the Blood/Plasma concentration ratio versus P_eff_ for peptides P1–P4 (Figure 8) demonstrates that P2 and P4 demonstrate higher values of these parameters, which are in line with the enhanced profiles of bioavailability of these peptides. 

The results from compartmental absorption also demonstrate that P1 (Figure S4), P2 (Figure S5), and P4 (Figure S7) have higher absorption in the jejunum (4.9, 13.4, and 13.4%, respectively) than P3 (Figure S6, 1.5%), while this peptide has an increased accumulation in the ascendent colon (17.2%) compared to other peptides (3.4, 1.1, and 0.7%, for peptides P1, P2, and P4, respectively). Therefore, we predicted that P3 would produce better results in HT-29 colon cancer cells than in MCF-7 and PC-3 prostate cancer cells.

Taken together, these results suggest that drug combinations using 5-FU in HT-29 colorectal cancer cells will result in enhanced anticancer effects than using DOXO and PTX in PC-3 prostate and MCF-7 breast cancer cells, respectively. Furthermore, these results also predict that due to the higher bioavailability and lower T_max_ of P2–P4, these peptides will be able to interact most strongly and faster with 5-FU, which can lead to synergistic interactions. As P3 is a conjugate of CPP6 (P1) and CFLG (P2) connected by a linker (PEG), we hypothesized this peptide would also result in enhanced drug transport and internalization of 5-FU into cancer cells than P1 and P2.

### 2.3. In Vitro Studies on the Anticancer Activity of Peptides P1–P4 Alone and Combined with Chemotherapeutic Agents

To further confirm our previous in silico predictions, we next evaluated all peptides, both alone and in combination with 5-FU, DOXO, and PTX, in HT-29, PC-3, and MCF-7 cancer cells, respectively. After finding the most promising drug combinations, the calculation of the combination index was performed using the Chou-Talalay method to assess for synergic interactions. 

#### 2.3.1. Anticancer Activity of Peptides P1–P4

**Figure 9:**
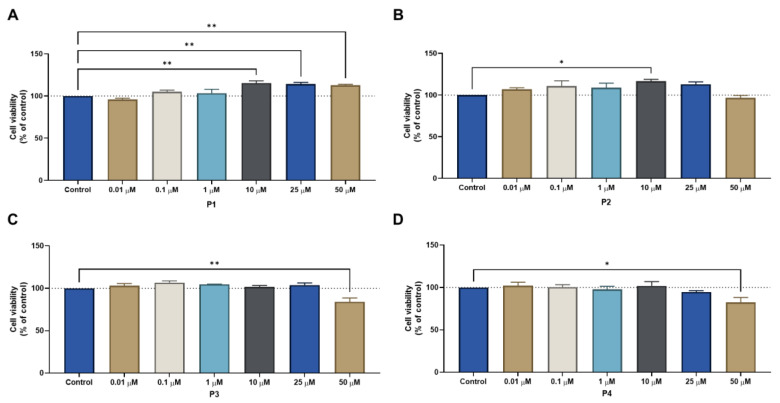
Cytotoxic results of MCF-7 after exposure to increasing concentrations of peptides (**A**) P1, (**B**) P2, (**C**) P3, and (**D**) P4 (0.01–50 μM) for 48 h. Control cells were treated with 0.01% DMSO (vehicle). Cell viability was obtained using the MTT assay, and the results are given as the mean ± SEM (*n* = 3). * Statistically significant vs. control (vehicle) at *p* < 0.05, ** statistically significant vs. control (vehicle) at *p* < 0.01.

**Figure 10:**
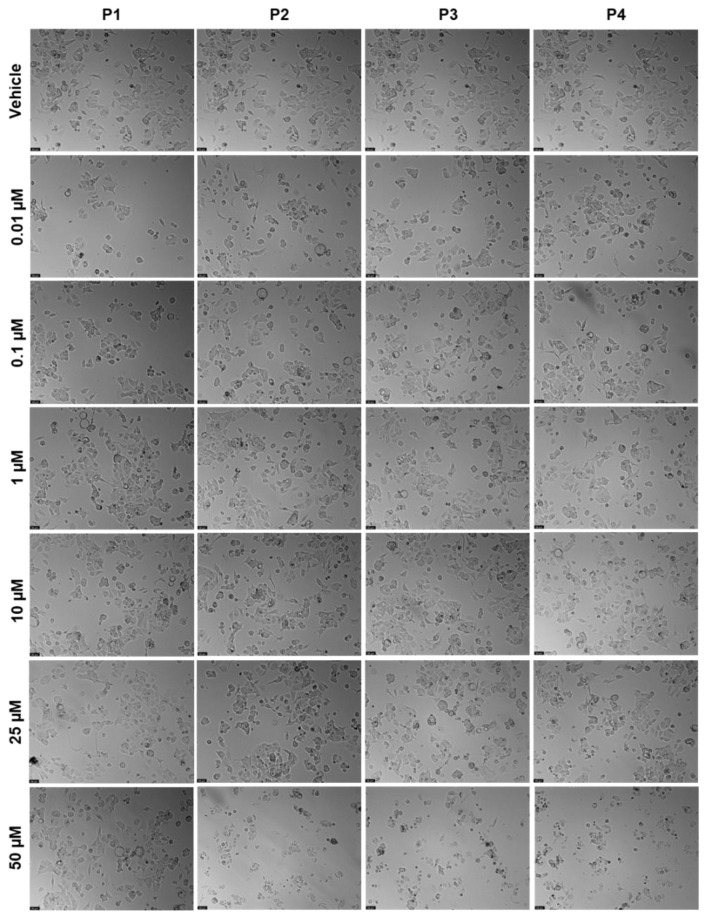
Morphological evaluation of MCF-7 after exposure to increasing concentrations of P1–P4 (0.01–100 μM) for 48 h. Control cells were treated with the vehicle (0.01% DMSO). These images are representative of three independent experiments. Scale bar is 200 μM.

First, the four peptides (P1–P4) were tested alone on three different cell lines (MCF-7, HT-29, and PC-3), using concentrations of 0.01, 0.1, 1, 10, 25, and 50 μM for 48 h. The results for the MCF-7 cell line regarding cell viability and morphological images are represented in Figure 9 and Figure 10, respectively. P1 and P2 had no effects on cell viability (Figure 9A,B, respectively), which made it impossible to obtain an IC_50_ value with the concentrations used. Meanwhile, P3 and P4 showed a statistically significant decrease in cell viability when cells were treated with the highest concentration (50 μM) (Figure 9C,D, respectively). However, this decrease was only about 20% of cell viability reduction, both for P3 and P4, so no IC_50_ could be obtained for these concentrations as well. These results were in accordance with the morphological analysis of MCF-7 cells treated with each peptide (Figure 10), where it was found that the number of MCF-7 cells decreased in the treatments with P2–P4 at a concentration of 50 µM (Figure 10).

**Figure 11:**
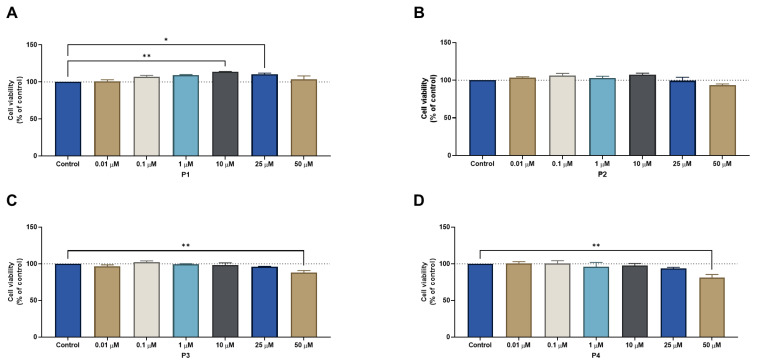
Cytotoxic results of HT-29 after exposure to increasing concentrations of peptides (**A**) P1, (**B**) P2, (**C**) P3, and (**D**) P4 (0.01–50 μM) for 48 h. Control cells were treated with 0.01% DMSO (vehicle). Cell viability was obtained using the MTT assay and the results are given as the mean ± SEM (*n* = 3). * Statistically significant vs. control (vehicle) at *p* < 0.05, ** statistically significant vs. drug alone at *p* < 0.01.

**Figure 12:**
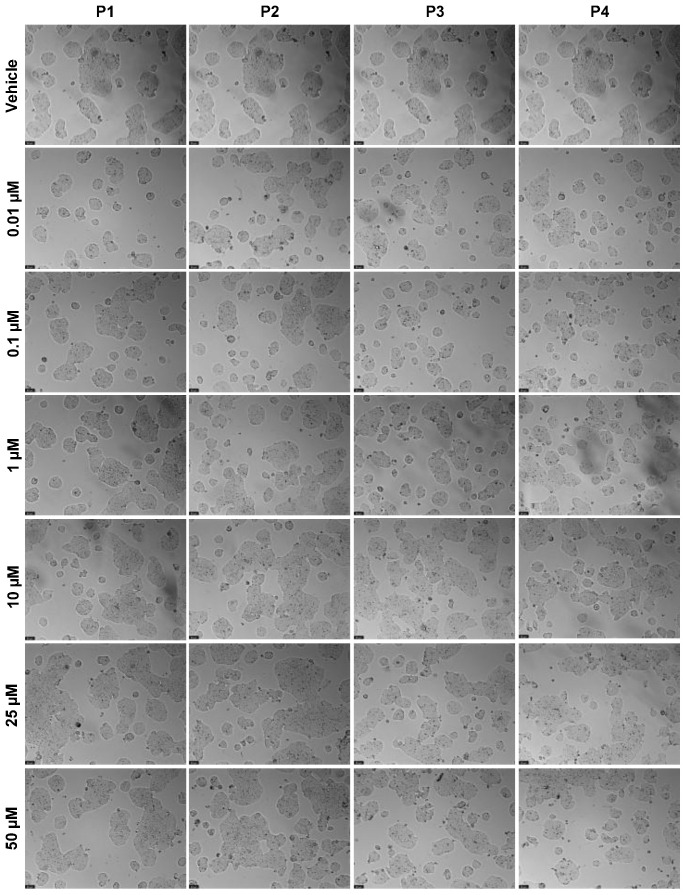
Morphological evaluation of HT-29 after exposure to increasing concentrations of P1–P4 (0.01–50 μM) for 48 h. Control cells were treated with the vehicle (0.01% DMSO). These images are representative of three independent experiments. Scale bar is 200 μM.

The results for the HT-29 cells regarding cell viability and morphological evaluation of cells from peptides tested alone at concentrations of 0.01, 0.1, 1, 10, 25, and 50 μM for 48 h can be found in Figure 11 and Figure 12, respectively. The results are similar to those obtained for MCF-7, with P1 and P2 having no significant effect on the reduction of cell viability (Figure 11A,B, respectively); on the other hand, P3 and P4 caused a significant decrease in cell viability but only at the highest concentration of 50 μM (Figure 11C,D, respectively). Likewise, no IC_50_ could be calculated for any peptide with the concentrations tested, meaning that it must be higher than 50 μM. The results of the morphological analysis (Figure 12) are in accordance with the MTT results and demonstrate a reduction in the number and size of cellular aggregates for treatments with P3 and P4 at a concentration of 50 µM.

**Figure 13:**
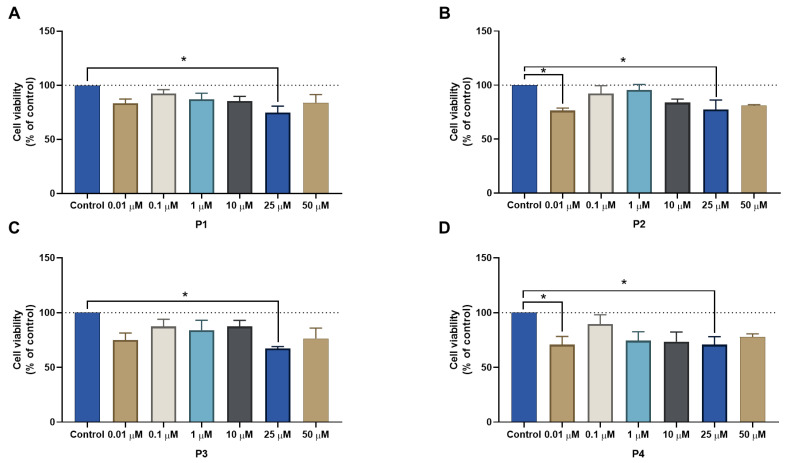
Cytotoxic results of PC-3 after exposure to increasing concentrations of peptides (**A**) P1, (**B**) P2, (**C**) P3, and (**D**) P4 (0.01–50 μM) for 48 h. Control cells were treated with 0.01% DMSO (vehicle). Cell viability was obtained using the MTT assay, and the results are given as the mean ± SEM (*n* = 3). * Statistically significant vs. control (vehicle) at *p* < 0.05.

**Figure 14:**
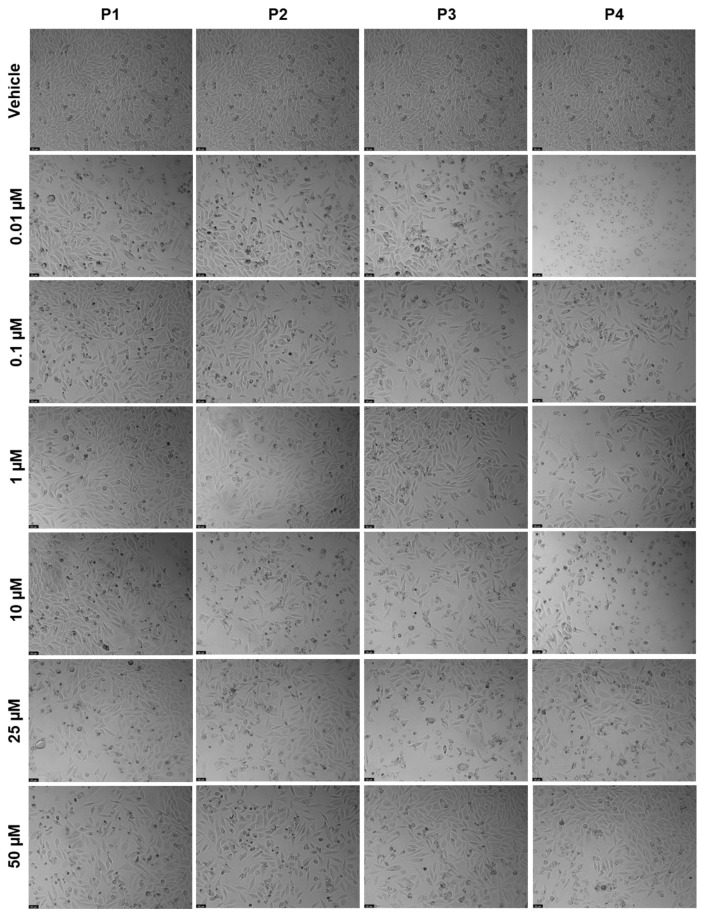
Morphological evaluation of PC-3 after exposure to increasing concentrations of P1–P4 (0.01–50 μM) for 48 h. Control cells were treated with the vehicle (0.01% DMSO). These images are representative of three independent experiments. Scale bar is 200 μM.

The following step was to assess the cytotoxicity of each peptide in the PC-3 prostate cells. Concentrations of 0.01, 0.1, 1, 10, 25, and 50 μM were tested for 48 h, and the results of cell viability and morphological evaluation images can be seen in Figure 13 and Figure 14, respectively. The results demonstrate that there was a significant decrease in cell viability for all peptides at the concentration of 25 μM (Figure 13); moreover, P2 and P4 also induced a significant reduction in the number of viable cells at the concentration of 0.01 μM (Figure 13B,C, respectively). Based on these results, no IC_50_ could be obtained for any peptide in this cell line. The results regarding morphological evaluation (Figure 14) are in accordance with the MTT results and demonstrate a reduction in the number of cells in the treatments with all peptides at a concentration of 25 µM.

**Figure 15:**
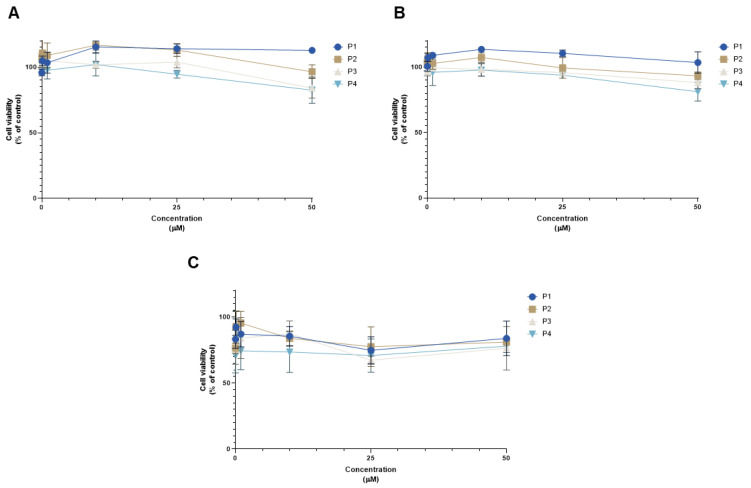
Comparison of cell viability results of (**A**) MCF-7, (**B**) HT-29, and (**C**) PC-3 cells after exposure to increasing concentrations of the peptides P1–P4 (0.01–50 μM) for 48 h. Control cells were treated with 0.01% DMSO (vehicle). Cell viability was obtained using the MTT assay, and the results are given as the mean ± SEM (*n* = 3).

The summary of the results regarding the viability of all cell lines treated with increasing concentrations of each peptide is represented in Figure 15. It can be concluded that P1 has the least influence on MCF-7 cell viability, while P3 together with P4 had the strongest influence on the viability of this cell line (Figure 15A). Regarding HT-29 cells, like MCF-7 cells, P1 had the smallest effect on cell viability, while P4 had the greatest (Figure 15B). Generally, for PC-3 cells, all the peptides had a comparable profile, with P4 having slightly lower cell viability values (Figure 15C).

These results demonstrate that peptides P1–P4 do not induce significant anticancer effects in HT-29 colon and MCF-7 breast cancer cell lines at lower concentrations (<25 µM). In the case of PC-3 cells, these peptides are also relatively safe for this cell line and do not induce a reduction of cell viability of more than 30%. Taken together, these results also support that these peptides have an acceptable safety profile, making them ideal candidates to increase drug transport and delivery into cells.

#### 2.3.2. Anticancer Efficacy of the Combination of Peptides P1–P4 with Chemotherapeutic Drugs

After finding that peptides P1–P4 do not demonstrate promising anticancer effects on these cell lines, we hypothesized that their combination with conventional chemotherapy could help increase the transport of such drugs into different cancer cells and therefore increase the anticancer potential of these antineoplastic agents. 

To do so, we combined increasing concentrations of peptides P1–P4 with conventional antineoplastic agents commonly used for breast, colon, and prostate cancer, namely PTX, 5-FU, and DOXO, respectively, at their IC_50_ (Table 3). As these drugs are widely used for cancer therapy, their IC_50_ value was obtained from the literature [27,28,29].

**Figure 16:**
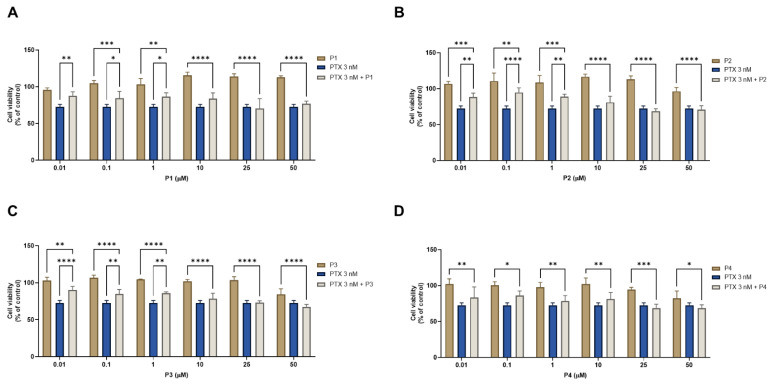
Cytotoxic results of MCF-7 after exposure to combinations of 3 nM of paclitaxel with increasing concentrations of the peptides (**A**) P1, (**B**) P2, (**C**) P3, and (**D**) P4 (0.01–50 μM) for 48 h. Control cells were treated with 0.01% DMSO (vehicle). Cell viability was obtained using the MTT assay, and the results are given as the mean ± SEM (*n* = 3). * Statistically significant vs. drug alone at *p* < 0.05, ** statistically significant vs. drug alone at *p* < 0.01, *** statistically significant vs. drug alone at *p* < 0.001, **** statistically significant vs. drug alone at *p* < 0.0001.

**Figure 17:**
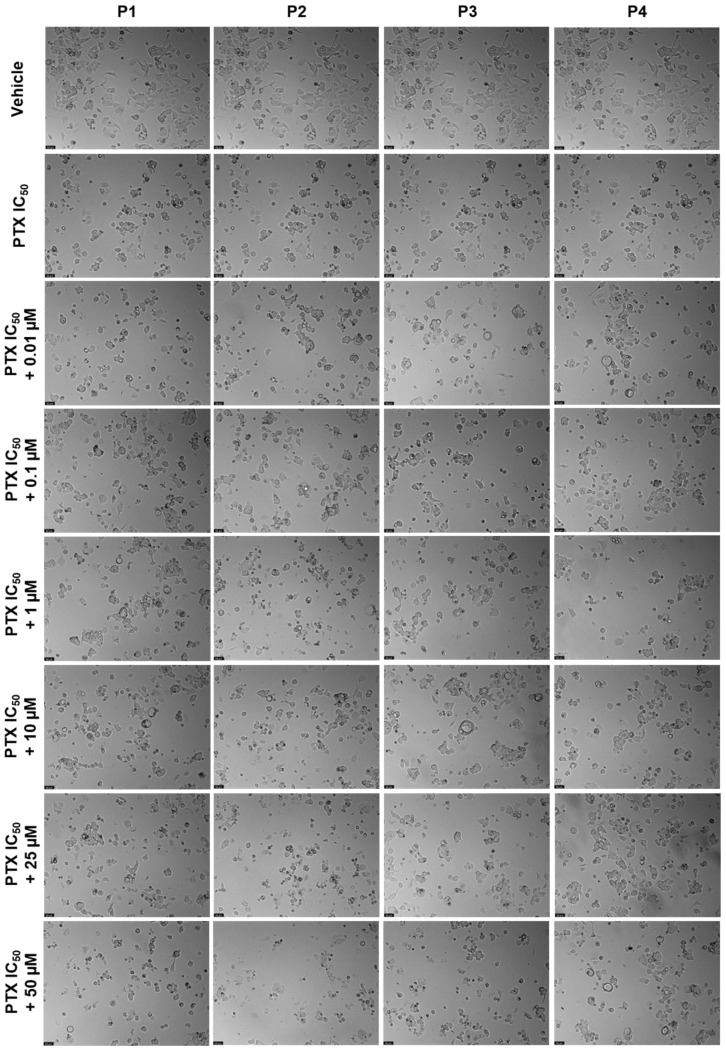
Morphological evaluation of MCF-7 after exposure to a combination of IC_50_ of PTX (3 nM) with increasing concentrations of P1–P4 (0.01–50 μM) for 48 h. Both substances were added at the same time. Control cells were treated with the vehicle (0.01% DMSO). These images are representative of three independent experiments. Scale bar is 200 μM.

To perform the combination studies in MCF-7 cells, 3 nM of PTX was combined with concentrations of 0.01, 0.1, 1, 10, 25, and 50 μM of each peptide. The results of cell viability of combinations against each compound alone and the morphological evaluation are represented in Figure 16 and Figure 17, respectively. For all peptides, the combination was more effective than the peptide alone at all ranges of concentrations (Figure 16). Meanwhile, when compared with PTX alone, the combinations were not as effective in decreasing MCF-7 cell viability, and the combined effect seems to reflect the anticancer activity of the chemotherapeutic agent; nevertheless, it is clear that increasing peptide concentrations also interfered with the combined effect. The results of the morphological analysis (Figure 17) are in accordance with these results and demonstrate a pronounced reduction in cell number and changes in the size and shape of MCF-7 cells after all treatments, compared to vehicle.

**Figure 18:**
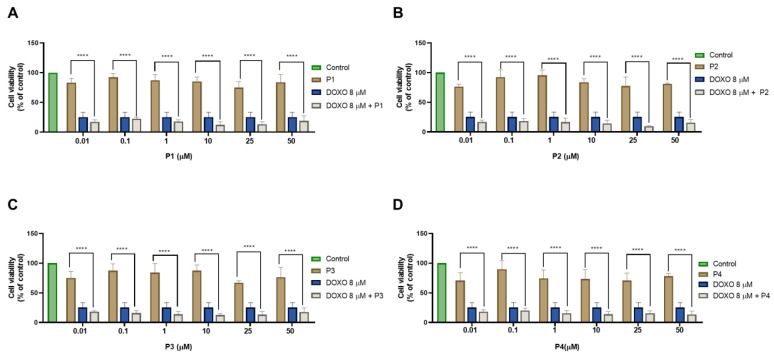
Cytotoxic results of PC-3 after exposure to combinations of 8 μM of doxorubicin with increasing concentrations of the peptides (**A**) P1, (**B**) P2, (**C**) P3, and (**D**) P4 (0.01–50 μM) for 48 h. Control cells were treated with 0.01% DMSO (vehicle). Cell viability was obtained using the MTT assay, and the results are given as the mean ± SEM (*n* = 3). **** Statistically significant vs. drug alone at *p* < 0.0001.

**Figure 19:**
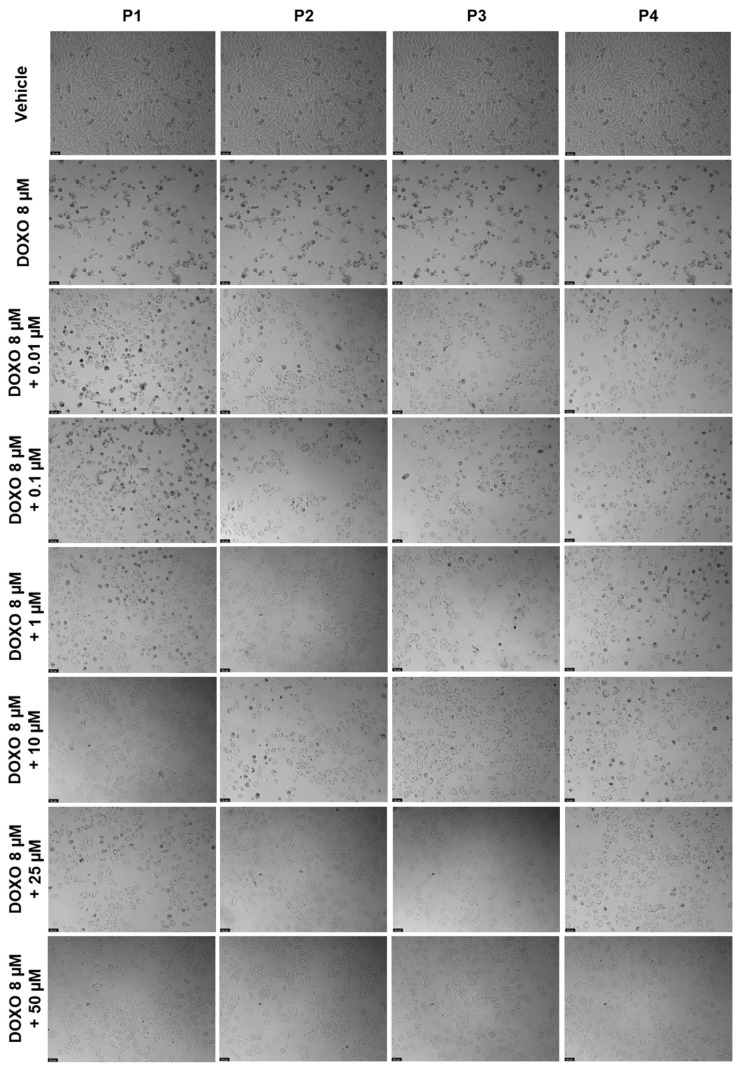
Morphological evaluation of PC-3 after exposure to a combination of 8 μM of DOXO with increasing concentrations of P1–P4 (0.01–50 μM) for 48 h. Both substances were added at the same time. Control cells were treated with the vehicle (0.01% DMSO). These images are representative of three independent experiments. Scale bar is 200 μM.

Combination studies were then performed in PC-3 cells treated with each peptide and DOXO. The results of cell viability and the morphological evaluation images from a combination of 8 μM of DOXO with increasing concentrations (0.01, 0.1, 1, 10, 25, and 50 μM) of each peptide and their comparison against DOXO and the peptides alone are shown in Figure 18 and Figure 19, respectively. All combinations induced a significant reduction of cell viability compared to each peptide alone (Figure 18); nevertheless, similar to the MCF-7 results, no significant results were found between the anticancer effect of DOXO alone and the combined pairs, demonstrating that the combined anticancer effect may be derived from the anticancer drug alone. These results are supported by the analysis of the cellular morphology represented in Figure 19, where cells become smaller, rounder, and fewer in number after treatments with DOXO and all drug combinations, compared to control cells.

**Figure 20:**
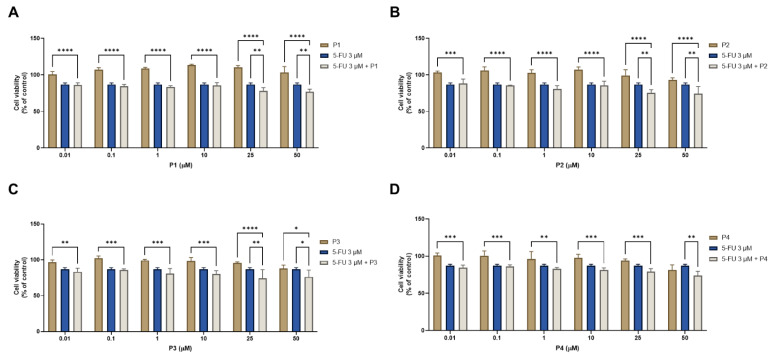
Cytotoxic results of HT-29 after exposure to combinations of 3 μM of 5-FU with increasing concentrations of the peptides (**A**) P1, (**B**) P2, (**C**) P3, and (**D**) P4 (0.01–50 μM) for 48 h. Control cells were treated with 0.01% DMSO (vehicle). Cell viability was obtained using the MTT assay, and the results are given as the mean ± SEM (*n* = 3). * Statistically significant vs. drug alone at *p* < 0.05, ** statistically significant vs. drug alone at *p* < 0.01, *** statistically significant vs. drug alone at *p* < 0.001, **** statistically significant vs. drug alone at *p* < 0.0001.

**Figure 21:**
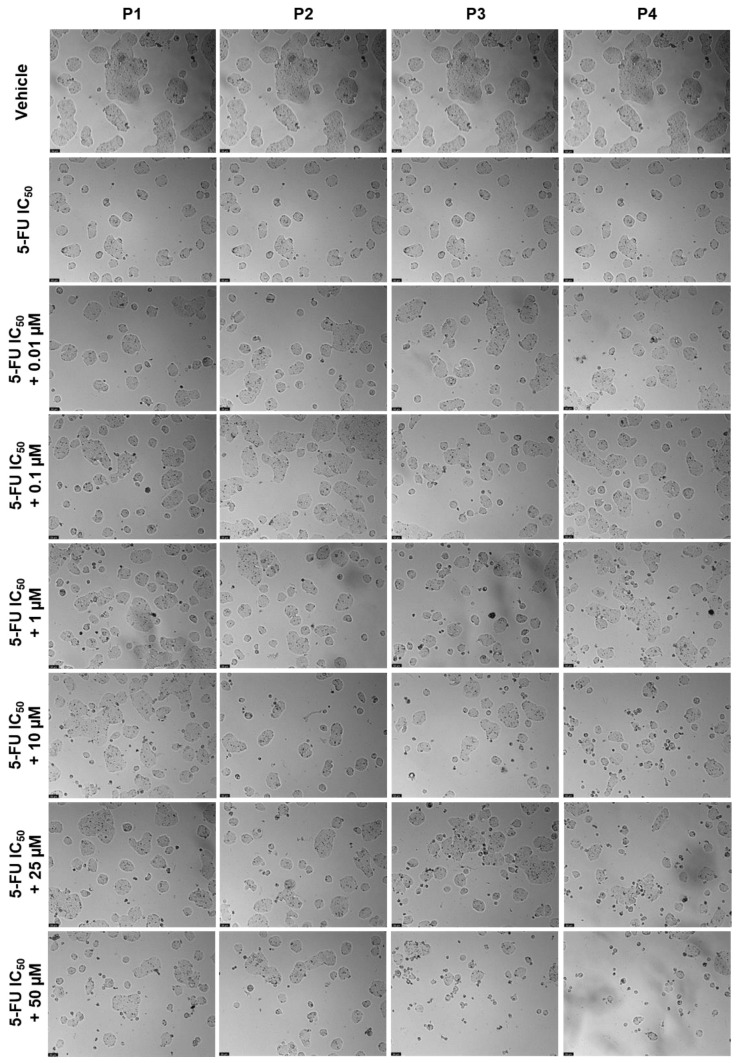
Morphological evaluation of HT-29 after exposure to a combination of the IC_50_ of 5-FU (3 μM) with increasing concentrations of P1–P4 (0.01–50 μM) for 48 h. Both substances were added at the same time. Control cells were treated with the vehicle (0.01% DMSO). These images are representative of three independent experiments. Scale bar is 200 μM.

Regarding HT-29 results, the cell viability graphs and the morphological evaluation images of the combination of 3 μM of 5-FU with 0.01, 0.1, 1, 10, 25, and 50 μM of each peptide for 48 h against 5-FU and the peptides alone can be seen in Figure 16 and Figure 17, respectively. All combinations had more significant effects on cell viability than the peptides alone (Figure 20), except for the combination of 50 μM of P4 and 5-FU, where the combined effect was better than only 5-FU alone (Figure 20D). Combinations of 5-FU with 25 and 50 μM of P1 (Figure 20A), P2 (Figure 20B), and P3 (Figure 20C) resulted in a higher reduction of HT-29 cell viability than both the corresponding peptides and 5-FU alone, demonstrating these peptides can be used to enhance the delivery of 5-FU into cancer cells when presented at higher concentrations. The results of the analysis of cell morphology (Figure 21) support these results and demonstrate a reduction in the number and size of cell aggregates after all treatments, compared to vehicle.

**Figure 22:**
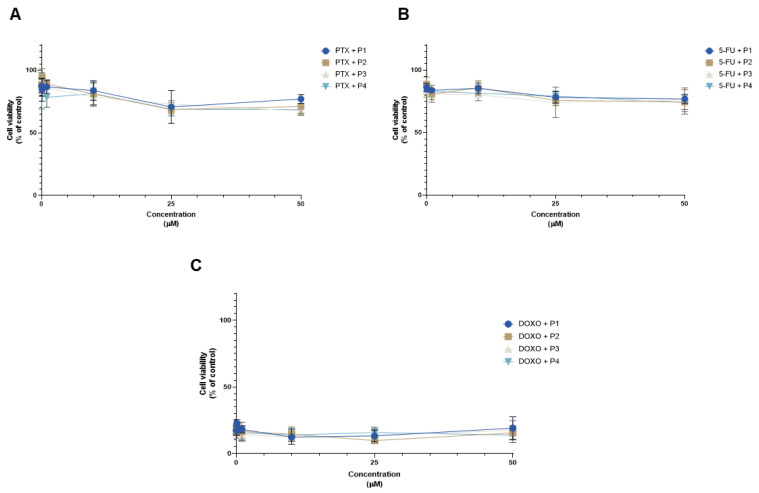
Comparison of the cell viability results of (**A**) MCF-7, (**B**) HT-29, and (**C**) PC-3 cells after exposure to combinations of chemotherapeutic drugs with increasing concentrations of the peptides P1–P4 (0.01–50 μM) for 48 h. Control cells were treated with 0.01% DMSO (vehicle). Cell viability was obtained using the MTT assay, and the results are given as the mean ± SEM (*n* = 3).

Figure 22 denotes the comparison of cell viability among all cell lines against the concentration of combinations for all peptides. There was practically no difference in values among the different peptides in MCF-7 cells (Figure 22A). Combining the different peptides with 5-FU showed similar effects on cell viability between themselves in HT-29 cells (Figure 22B). Comparing the combinations with each other in PC-3 cells shows a relatively uniform effect among them, with the DOXO and P2 combination being marginally stronger for higher concentrations of P2 (Figure 22C).

Taken together, the results previously obtained in MCF-7 breast cancer cells and PC-3 prostate cancer cells demonstrate that the combination of peptides P1–P4 is not advantageous over the use of the chemotherapeutic agents alone. Nevertheless, in the case of HT-29 colorectal cancer cells, it was possible to verify that the combination of P1–P3 with 5-FU at higher concentrations (25 and 50 µM) induces higher cytotoxicity than 5-FU and each peptide alone, demonstrating that these combinations can be promising for increasing the efficacy of colorectal cancer therapy.

Furthermore, these results are in line with those obtained through the in silico simulations, where we predicted that 5-FU would have an enhanced ability to enter cancer cells and to exert more cytotoxic effects than DOXO and PTX, as well as higher bioavailability, making it more available to interact with peptides.

#### 2.3.3. Combination Index Evaluation in the Combination of Peptides P1–P4 with 5-FU in HT-29 Colorectal Cancer Cells

After finding the most promising drug combinations based on the cell viability results and the software CompuSyn, we finally assessed the combination synergism. This was only performed for the combinations of 5-FU plus each peptide, since the combinations tested in MCF-7 and PC-3 cancer cells did not present significant combined anticancer effects.

**Figure 23:**
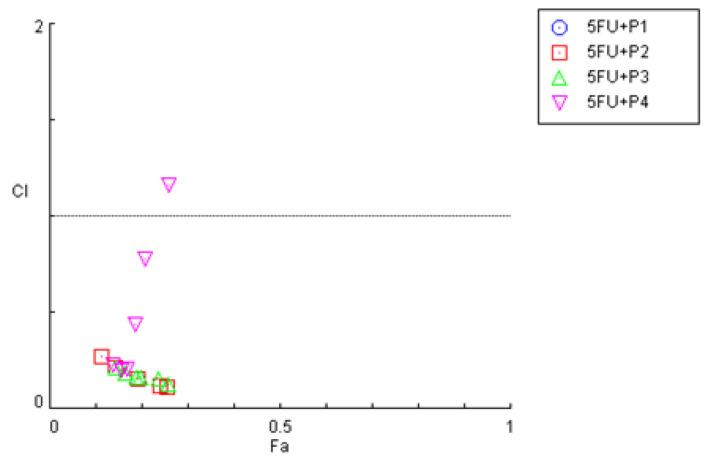
Effect level–combination index (Fa-CI) plot obtained for HT-29 cells after exposure to the combination of 5-FU with increasing concentrations of the peptides P1–P4, obtained using the Chou–Talalay method. CI < 1—synergism; CI = 1—additivity CI > 1—antagonism.

The combination index (CI) plot for the drug combinations of 5-FU plus peptides P1–P4 can be seen in Figure 23; in this plot are represented the individual values of CI vs the fractional effect (Fa). CI values higher than one indicate antagonism, equal to one indicate additivity, and lower than one indicate synergism. An Fa value equal to zero indicates no cell death whereas one indicates complete cell death. These results are also summarized in Table 4.

The combination index plot obtained for the combinations in HT-29 cells demonstrates that most CI values obtained for the combination of 5-FU with each peptide are below one, which indicates that most of the combined pairs present synergic interactions. Nevertheless, in the combination of 5-FU with P4, some CI values are closer to additivity or even antagonism, which is in accordance with the previous MTT results obtained in Figure 9.

Analyzing Table 4, no CI values were possible to calculate for the combination of P1 + 5-FU. All the combinations of 5-FU with P2–P4 demonstrate CI values of synergism except for the combination of 5-FU with 50 μM of P4, which was antagonistic. In fact, the CI values for the combination of 5-FU and P4 tend to increase with the increaseinf P4 concentration, which indicates that this combination gets worse with the increasing addition of peptide.

Taken together, these results are in line with the in silico predictions where it was found that P2, P3, and P4 had greater bioavailability compared to P1. Therefore, we believe that these peptides are more likely to interact with 5-FU and further increase the delivery of this drug into cells. Moreover, as we demonstrated that these peptides have a lower T_max_ than P1, we also believe that these peptides interact faster and produce a rapid therapeutic effect when combined with 5-FU in these cells, resulting in synergistic interactions. 

These results demonstrate that in silico studies may be a viable complement to predict the cytotoxic activity of chemotherapeutic agents and peptides, both alone and in combination, for in vitro studies using cancer cells.

## 3. Materials and Methods

### 3.1. Cell Lines and Culture Conditions

MCF-7 (ATCC^®^ HTB-22) breast, HT-29 (ATCC^®^ HTB-38) colon, and PC-3 (ATCC^®^ CRL-1435) cancer cell lines were obtained from the American Type Culture Collection (ATCC, American Type Culture Collection, Manassas, VA, USA). MCF-7 and PC-3 cells were cultured in Dulbecco’s Modified Eagle Medium (DMEM, Merck KGaA, Darmstadt, Germany) and HT-29 cells in McCoy’s (Merck KGaA, Darmstadt, Germany) cell culture medium, which contained 10% fetal bovine serum (FBS, Merck KGaA, Darmstadt, Germany) and 1% of a mixture of penicillin G and streptomycin (1000 U/mL; 10 mg/mL, Merck KGaA, Darmstadt, Germany). Mycoplasma-free cultures were kept in a humidified atmosphere of 95% air and 5% CO_2_ at 37 °C.

### 3.2. Peptides and Drugs

All peptides were previously synthesized and purified by our group [16] and were dissolved in dimethyl sulfoxide (DMSO) at 100 mM as a stock solution. These peptides were diluted to obtain the desired final concentrations with the respective culture medium before use. Paclitaxel (PTX, cat. no. 1097, Tocris Bioscience, Bristol, UK), 5-fluorouracil (5-FU, cat. no. F6627, Merck KGaA, Darmstadt, Germany), and doxorubicin (DOXO, cat. no. 15007, Cayman Chemical, Ann Arbor, MI, USA) were used as reference drugs in MCF-7, HT-29, and PC-3 cell lines, respectively, and were dissolved in DMSO. Drugs were diluted to their final concentrations with their respective culture mediums before use.

### 3.3. Cell Treatment and Viability Assay

The MTT experiment was conducted to assess the antiproliferative effect of each peptide alone and in combination with chemotherapeutic drugs. MCF-7, HT-29, and PC-3 cancer cells were seeded on 96-well plates at a density of 10,000 cells per well, for a volume of 200 µL. On the next day, the cell medium was removed, and cells were exposed to serial dilutions of each peptide alone (0.01 µM–50 µM) or combined with each reference drug (IC_50_, obtained in our laboratory or from literature) for 48 h. Control cells were treated with vehicle (0.1% DMSO). After this period, cells were incubated with 100 µL of the 3-(4,5-dimethylthiazol-2-yl)-2,5-diphenyltetrazolium bromide (MTT, cat. no. M5655, Merck KGaA, Darmstadt, Germany) solution at a concentration of 0.5 mg/mL for 3 h, protected from light. During this time, MTT was metabolized by living cells to purple formazan crystals which then were solubilized with 100 µL DMSO. Absorbance was measured at 570 nm using a microplate reader (Tecan Infinite M200, Tecan Group Ltd., Männedorf, Switzerland). All conditions were performed three times independently, in triplicate.

### 3.4. Cell Morphology Visualization

Cell morphological analysis was assessed after each treatment and before the MTT assays using a Leica DMI 6000B microscope equipped with a Leica DFC350 FX camera. Images were then analyzed with the Leica LAS X imaging software (v3.7.4).

### 3.5. Synergistic Effect Analysis

To quantify the drug interaction between the chemotherapeutic drugs and each peptide, the Chou-Talalay equation and CompuSyn software (version 1.0; ComboSyn, Paramus, NJ, USA) were used to estimate the Combination Index (CI) [25]. The CI was used to determine the types of drug interactions, where CI < 1 indicates synergism, CI = 1 indicates additivity, and CI > 1 represents antagonism. The simulations were performed using a non-fixed ratio of doses with a fixed concentration of chemotherapeutic drug and variable concentrations of each peptide.

### 3.6. Statistical Analysis

All assays were performed in triplicate with at least three independent experiments. All data were reported as mean ± standard error of the mean (SEM). Statistical analysis was carried out using GraphPad Prism, version 9.0 (San Diego, CA, USA). Statistical analysis was performed using an ordinary one-way ANOVA, with Dunnett’s multiple coparisons test. *P*-values < 0.05 were considered statistically significant.

## 4. Conclusions

Although in silico approaches represent a relatively new avenue of inquiry, these studies are starting to be used widely in studies to predict how drugs may interact and act with the organism and against cancer cells. In this study, we hypothesized that peptides P1–P4 could be used in combination with chemotherapeutic agents to enhance their delivery into different cancer cells. We performed in silico simulations to determine the PK profile of each peptide and chemotherapeutic agent, and based on these results, we made assumptions on the possible anticancer effect of these compounds in combination. We found 5-FU and P2–P4 to have the most promising PK profiles among all compounds simulated. We next evaluated each drug combination using in vitro assays and, in line with the in silico results, have found that higher concentrations of P2–P4 combined with 5-FU in HT-29 colorectal cancer cells resulted in enhanced cytotoxic effects than each molecule alone, characterized by synergism. Taken together, these results demonstrate that in silico approaches can be a promising auxiliary tool for the prediction of the interaction between drugs in combination and that these peptides can be used in combination with 5-FU to enhance the delivery of this drug into cancer cells for colorectal cancer therapy. In the future, these peptides should be further investigated for the determination of their exact mechanism of action in cancer cells and how they act synergistically with chemotherapeutic agents. Complementary assays should also be performed to confirm the efficacy of these peptides in the delivery of chemotherapeutic agents into cancer cells and to determine their safety against normal tissues. Moreover, since these peptides are biodegradable, their effect must be carefully studied in vivo to determine if they are still effective in more advanced cancer models in the delivery of anticancer agents.

## Figures and Tables

**Table 1:** Summary of simulated mean plasma PK parameters for GastroPlus^®^ simulations of 5-FU, DOXO, and PTX. The pharmacokinetic parameters are %F (% bioavailability), which is the percent of drug that reached the systemic circulation, T_max_, C_max_, and AUC (area under the curve).

	5-FU	DOXO	PTX
Blood/Plasma Conc. Ratio	1.2	1.09	0.67
Clearance, CL (L/h)	47.18	3.31	14.46
Central compartment volume, V_c_ (L/Kg)	0.38	6.86	3.11
Elimination half-life, T_1/2_ (h)	0.39	100.56	10.44
Effective permeability, P_eff_ (cm/s × 10^4^)	2.81	0.26	0.21
Fraction absorbed (Fa%), F_a_ (%)	98.60	53.4	7.23
Fraction of dose passing into the portal vein, F_Dp_ (%)	98.57	51.12	7.19
F (%)	55.66	49.40	5.47
C_max_ (μg/mL)	0.34	0.044	0.0058
T_max_ (h)	0.88	24	15.28
AUC_0–∞_ (μg-h/mL)	0.59	0.91	0.47
AUC_0–24h_ (μg-h/mL)	0.58	0.91	0.11
C_max Liver_ (μM/mL)	0.51	0.077	0.0075

**Table 2:** Summary of simulated mean plasma PK parameters for GastroPlus^®^ simulation of P1–P4.

	P1	P2	P3	P4
Blood/Plasma Conc. Ratio	0.73	0.79	0.68	1.03
CL (L/h)	14.73	15.57	26.7	15.05
V_c_ (L/Kg)	0.59	0.39	0.43	0.36
T_1/2_ (h)	1.94	1.22	0.78	1.16
P_eff_ (cm/s × 10^4^)	0.071	0.24	0.050	0.25
F_a_ (%)	20.26	43.14	34.45	39.52
F_Dp_ (%)	17.34	41.94	31.63	38.77
F (%)	13.15	32.77	17.73	37.45
C_max_ (μg/mL)	0.056	0.21	0.020	0.21
T_max_ (h)	4.24	2.8	3.84	2.72
AUC_0–∞_ (μg-h/mL)	0.58	1.05	0.41	1.08
AUC_0–24h_ (μg-h/mL)	0.45	1.05	0.32	1.08
C_max Liver_ (μM/mL)	0.069	0.26	0.029	0.25

**Table 3:** IC_50_ values of the antineoplastic agents PTX, 5-FU, and DOXO obtained from the literature and used in this study for combination treatments.

Compounds	IC_50_	Cell Line	Reference
PTX	3 nM	MCF-7	[27]
5-FU	3 µM	HT-29	[28]
DOXO	8 µM	PC-3	[29]

**Table 4:** Fractional effect (Fa) and CI values of peptides and 5-FU (3 μM) combinations for 48 h in HT-29 cells. C1 < 1 represents synergism, CI = 1 represents additivity, and C1 > 1 represents antagonism. The fractional effect shows the degree of cell death, with zero being no cellular death and one being total cellular death. CI in bold indicates drug pairs that are synergic.

Peptide (μM)	P1		P2		P3		P4	
	Fa	CI	Fa	CI	Fa	CI	Fa	CI
0.01	0.1374	NaN	0.1146	**0.26947**	0.1664	**0.18337**	0.1566	**0.19586**
0.1	0.1539	NaN	0.1427	**0.21549**	0.144	**0.21336**	0.1365	**0.22910**
1	0.1617	NaN	0.1922	**0.15691**	0.1894	**0.16051**	0.1695	**0.20947**
10	0.1456	NaN	0.1425	**0.21644**	0.1983	**0.16151**	0.1858	**0.43931**
25	0.2182	NaN	0.2423	**0.12144**	0.2577	**0.12611**	0.2081	**0.77552**
50	0.2309	NaN	0.2548	**0.1151**	0.2375	**0.15712**	0.2591	1.16511

## Data Availability

Not applicable.

## Supplementary Materials

The supporting information can be downloaded at: https://www.mdpi.com/article/10.3390/ijms24010069/s1.

## References

[1] Chabner B.A., Roberts T.G. (2005). Chemotherapy and the War on Cancer. Nat. Rev. Cancer.

[2] Guidotti G., Brambilla L., Rossi D. (2017). Cell-Penetrating Peptides: From Basic Research to Clinics. Trends Pharmacol. Sci..

[3] Böhme D., Beck-Sickinger A.G. (2015). Drug Delivery and Release Systems for Targeted Tumor Therapy. J. Pept. Sci..

[4] Knop K., Hoogenboom R., Fischer D., Schubert U.S. (2010). Poly(Ethylene Glycol) in Drug Delivery: Pros and Cons as Well as Potential Alternatives. Angew. Chem. Int. Ed..

[5] Vivès E., Schmidt J., Pèlegrin A. (2008). Cell-Penetrating and Cell-Targeting Peptides in Drug Delivery. Biochim. Biophys. Acta-Rev. Cancer.

[6] Xie J., Bi Y., Zhang H., Dong S., Teng L., Lee R.J., Yang Z. (2020). Cell-Penetrating Peptides in Diagnosis and Treatment of Human Diseases: From Preclinical Research to Clinical Application. Front. Pharmacol..

[7] Gräslund A., Madani F., Lindberg S., Langel Ü., Futaki S. (2011). Mechanisms of Cellular Uptake of Cell-Penetrating Peptides. J. Biophys..

[8] Derossi D., Calvet S., Trembleau A., Brunissen A., Chassaing G., Prochiantz A. (1996). Cell Internalization of the Third Helix of the Antennapedia Homeodomain Is Receptor-Independent. J. Biol. Chem..

[9] Matsuzaki K., Yoneyama S., Murase O., Miyajima K. (1996). Transbilayer Transport of Ions and Lipids Coupled with Mastoparan X Translocation. Biochemistry.

[10] Pouny Y., Rapaport D., Mor A., Nicolas P., Shai Y., Mor A., Nicolas P. (1992). Interaction of Antimicrobial Dermaseptin and Its Fluorescently Labeled Analogues with Phospholipid Membranes. Biochemistry.

[11] Lee M.T., Hung W.C., Chen F.Y., Huang H.W. (2005). Many-Body Effect of Antimicrobial Peptides: On the Correlation between Lipid’s Spontaneous Curvature and Pore Formation. Biophys. J..

[12] Jones A.T. (2007). Macropinocytosis: Searching for an Endocytic Identity and Role in the Uptake of Cell Penetrating Peptides. J. Cell. Mol. Med..

[13] Mayor S., Pagano R.E. (2007). Pathways of Clathrin-Independent Endocytosis. Nat. Rev. Mol. Cell Biol..

[14] Duarte D., Nunes M., Ricardo S., Vale N. (2022). Combination of Antimalarial and CNS Drugs with Antineoplastic Agents in MCF-7 Breast and HT-29 Colon Cancer Cells: Biosafety Evaluation and Mechanism of Action. Biomolecules.

[15] Duarte D., Vale N. (2021). Synergistic Interaction of CPP2 Coupled with Thiazole Derivates Combined with Clotrimazole and Antineoplastic Drugs in Prostate and Colon Cancer Cell Lines. Int. J. Mol. Sci..

[16] Yang V., Pedrosa S.S., Fernandes R., Maurício A.C., Koksch B., Gärtner F., Amorim I., Vale N. (2019). Synthesis of PEGylated Methotrexate Conjugated with a Novel CPP6, in Sillico Structural Insights and Activity in MCF-7 Cells. J. Mol. Struct..

[17] Gomez J.A., Chen J., Ngo J., Hajkova D., Yeh I.J., Gama V., Miyagi M., Matsuyama S. (2010). Cell-Penetrating Penta-Peptides (CPP5s): Measurement of Cell Entry and Protein-Transduction Activity. Pharmaceuticals.

[18] Correia C., Xavier C.P.R.R., Duarte D., Ferreira A., Moreira S., Vasconcelos M.H., Vale N. (2020). Development of Potent CPP6–Gemcitabine Conjugates against Human Prostate Cancer Cell Line (PC-3). RSC Med. Chem..

[19] Lin L., Wong H. (2017). Predicting Oral Drug Absorption: Mini Review on Physiologically-Based Pharmacokinetic Models. Pharmaceutics.

[20] Agoram B., Woltosz W.S., Bolger M.B. (2001). Predicting the Impact of Physiological and Biochemical Processes on Oral Drug Bioavailability. Adv. Drug Deliv. Rev..

[21] Ferreira A., Lapa R., Vale N. (2021). Permeability of Gemcitabine and PBPK Modeling to Assess Oral Administration. Curr. Issues Mol. Biol..

[22] Santos J., Lobato L., Vale N. (2021). Clinical Pharmacokinetic Study of Latrepirdine via in Silico Sublingual Administration. Silico Pharmacol..

[23] Ferreira A., Moreira S., Lapa R., Vale N. (2021). Permeability Evaluation of Gemcitabine-CPP6 Conjugates in Caco-2 Cells. ADMET DMPK.

[24] Ferreira A., Martins H., Oliveira J.C., Lapa R., Vale N. (2021). In Silico Pharmacokinetic Study of Vancomycin Using PBPK Modeling and Therapeutic Drug Monitoring. Curr. Drug Metab..

[25] Chou T.C. (2010). Drug Combination Studies and Their Synergy Quantification Using the Chou-Talalay Method. Cancer Res..

[26] Bottens R.A., Yamada T. (2022). Cell-Penetrating Peptides (CPPs) as Therapeutic and Diagnostic Agents for Cancer. Cancers.

[27] Duarte D., Vale N. (2020). New Trends for Antimalarial Drugs: Synergism between Antineoplastics and Antimalarials on Breast Cancer Cells. Biomolecules.

[28] Duarte D., Cardoso A., Vale N. (2021). Synergistic Growth Inhibition of HT-29 Colon and MCF-7 Breast Cancer Cells with Simultaneous and Sequential Combinations of Antineoplastics and CNS Drugs. Int. J. Mol. Sci..

[29] Orzechowska E.J., Girstun A., Staron K., Trzcinska-Danielewicz J. (2015). Synergy of BID with Doxorubicin in the Killing of Cancer Cells. Oncol. Rep..

