# Supplementary material for: Prediction of Drug Synergism between Peptides and Antineoplastic Drugs Paclitaxel, 5-Fluorouracil, and Doxorubicin Using In Silico Approaches
Source: Int J Mol Sci. 2022 Dec 21;24(1):69. doi: 10.3390/ijms24010069 (PMC9820768; doi:10.3390/ijms24010069)
Supplement: Supplementary file 1 [file ijms-24-00069-s001.zip › ijms-2059829-supplementary.pdf]

Supplementary Materials

Prediction of Drug Synergism between Peptides and Antineoplastic Drugs Paclitaxel, 5-Fluorouracil and Doxorubicin using In Silico Approaches

Nuno Vale [1,2,3,*], Mariana Pereira [1,3,4], Joana Santos [1,3], Catarina Moura [1,3], Lara Marques [1,3] and Diana Duarte [1,3]

[1] OncoPharma Research Group, Center for Health Technology and Services Research (CINTESIS), Rua Doutor Plácido da Costa, 4200-450 Porto, Portugal
[2] Department of Community Medicine, Health Information and Decision (MEDCIDS), Faculty of Medicine, University of Porto, Alameda Professor Hernâni Monteiro, 4200-319 Porto, Portugal
[3] CINTESIS@RISE, Faculty of Medicine, University of Porto, Alameda Professor Hernâni Monteiro, 4200-319, Porto, Portugal
[4] Institute of Biomedical Sciences Abel Salazar (ICBAS), University of Porto, Rua Jorge Viterbo Ferreira, 228, 4050-313 Porto, Portugal
* Correspondence: nunovale@med.up.pt

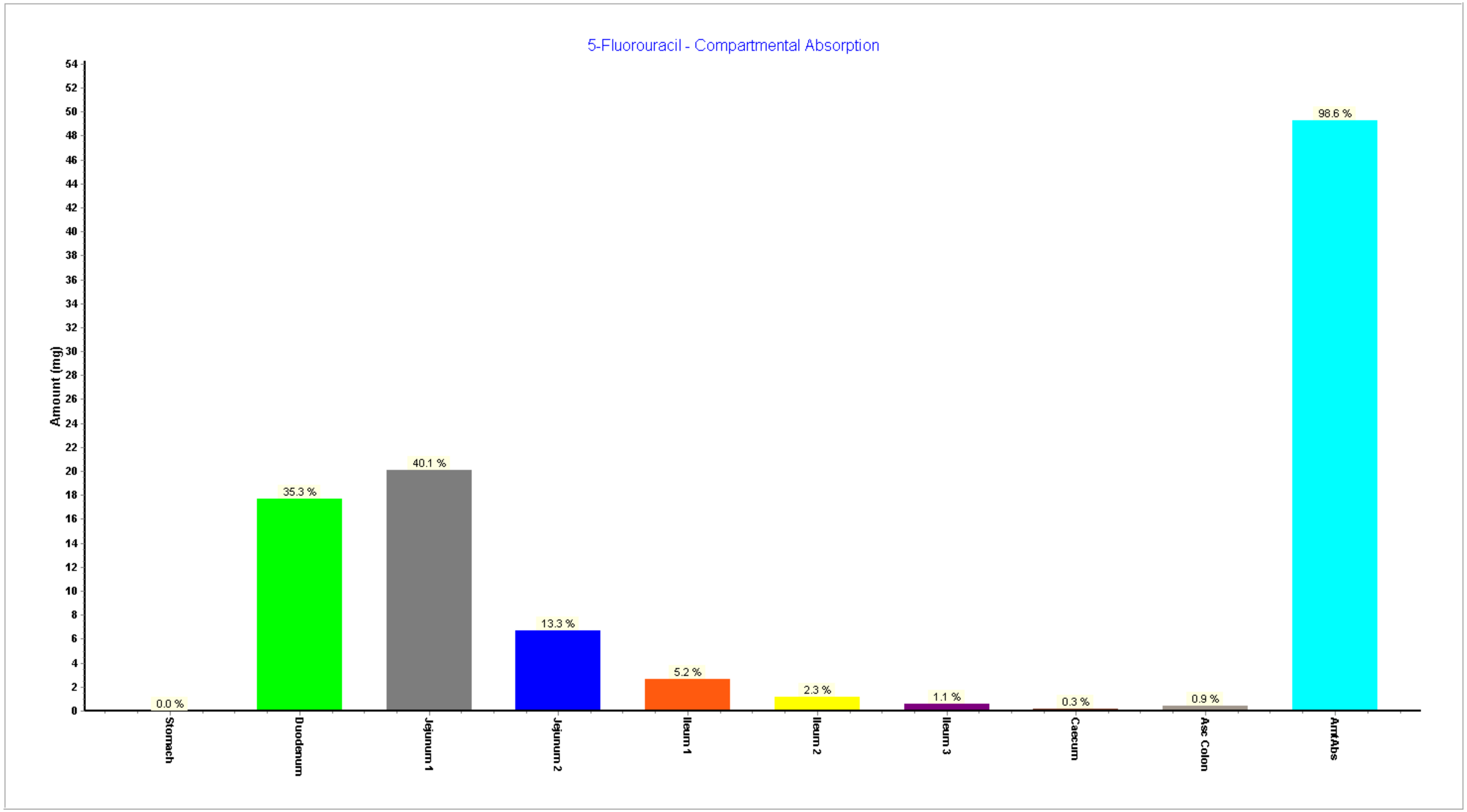

Figure S1. Compartmental absorption of 5-FU generated using the GastroPlus® software.

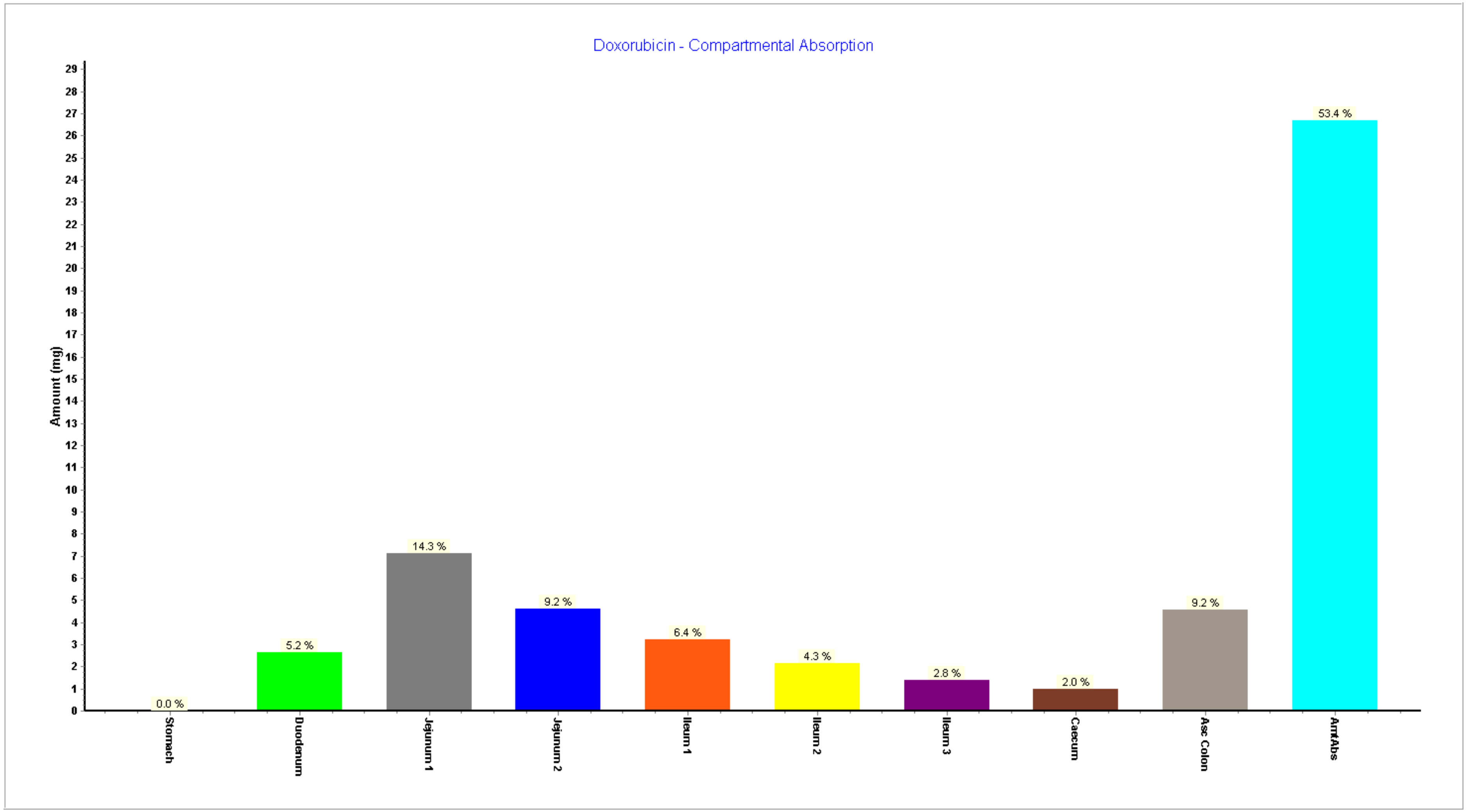

Figure S2. Compartmental absorption of DOXO generated using the GastroPlus® software.

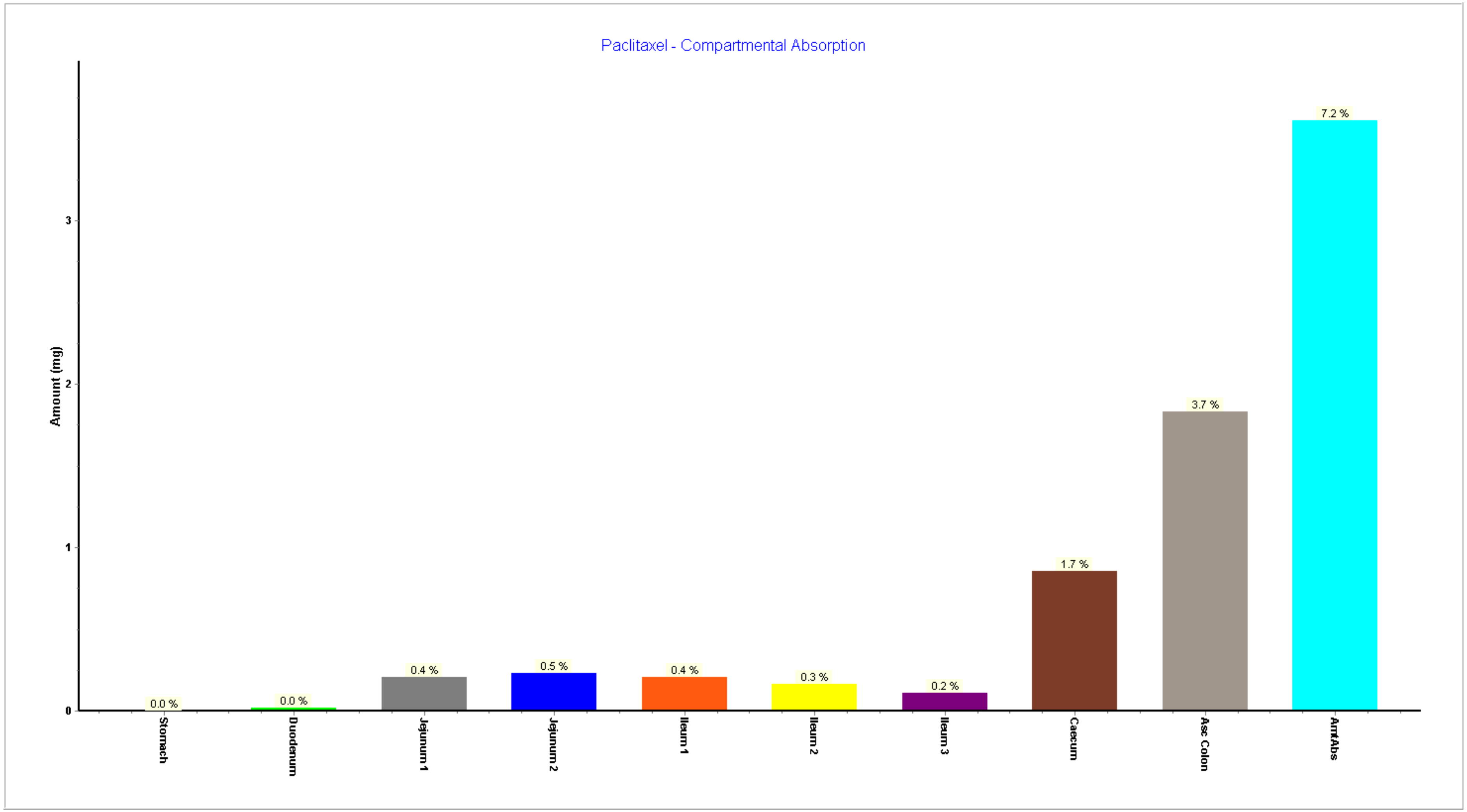

Figure S3. Compartmental absorption of PTX generated using the GastroPlus® software.

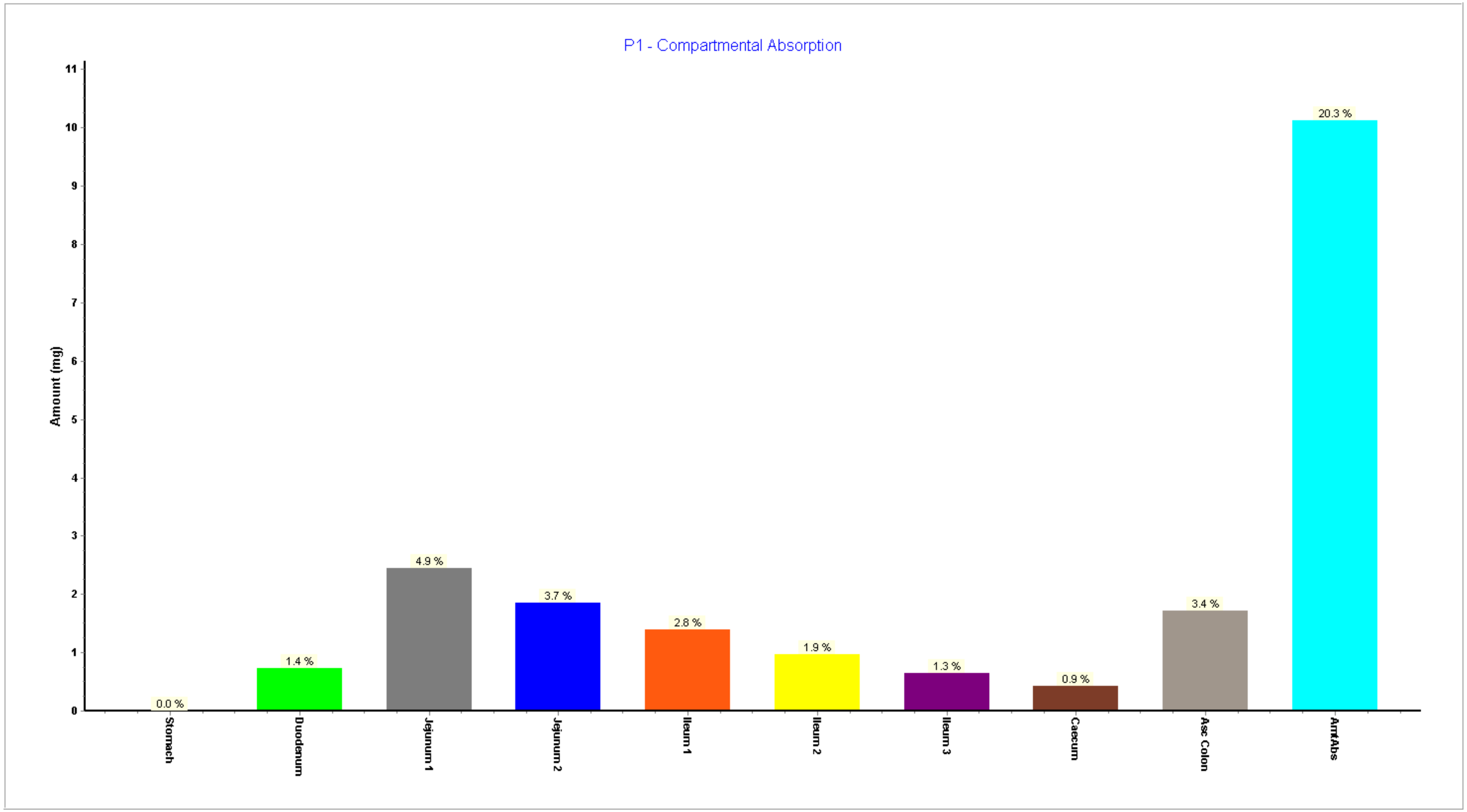

Figure S4. Compartmental absorption of P1 generated using the GastroPlus® software.

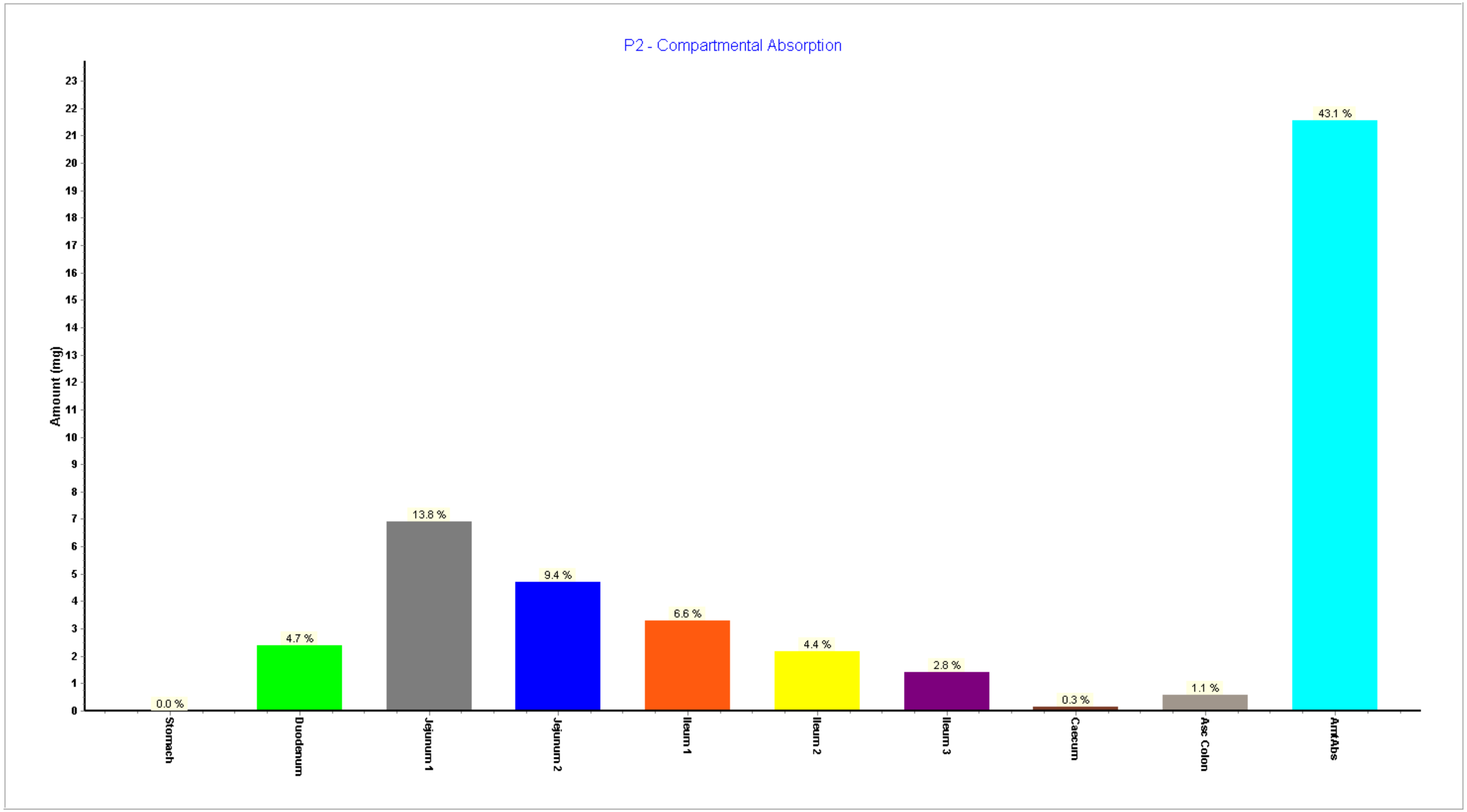

Figure S5. Compartmental absorption of P2 generated using the GastroPlus® software.

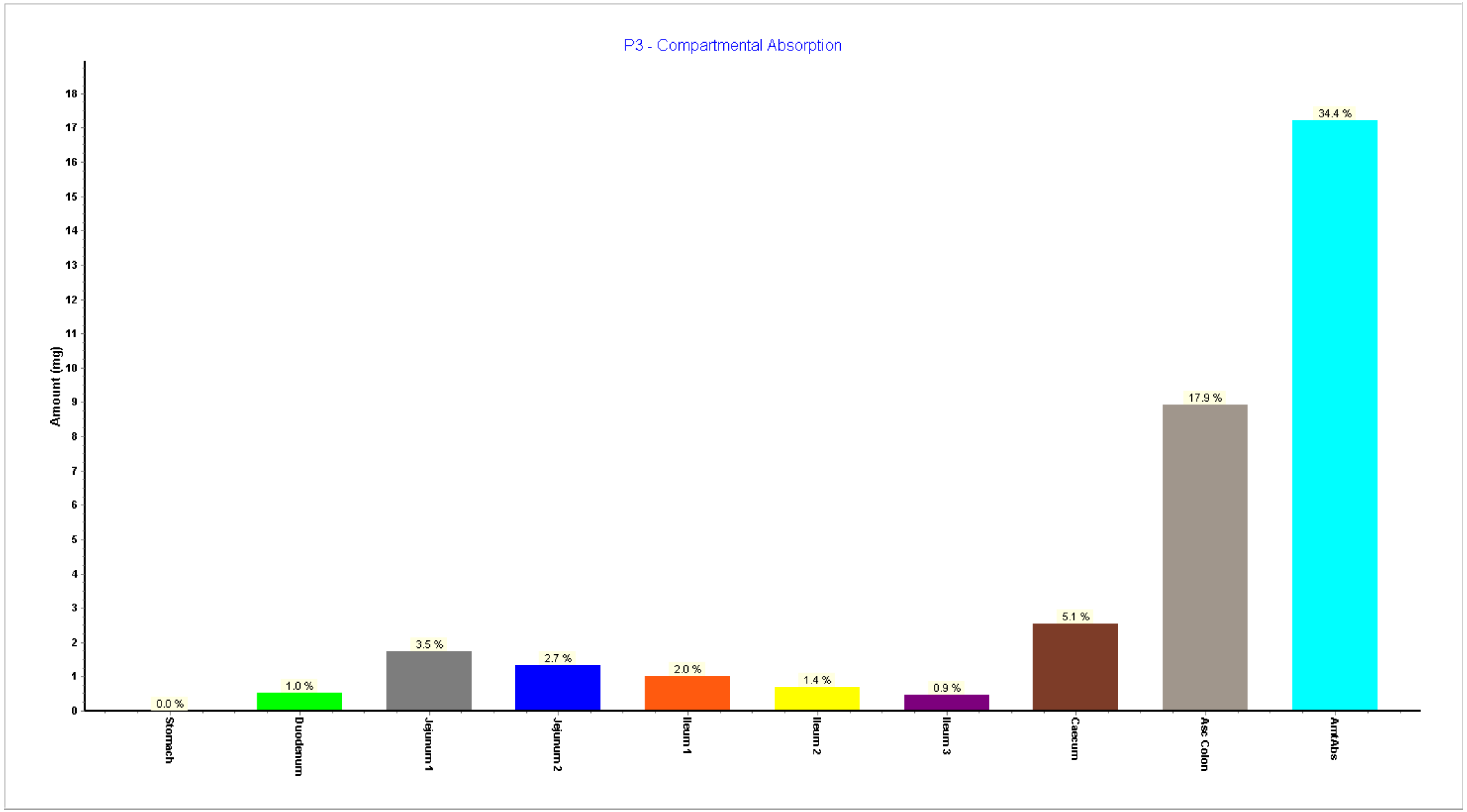

Figure S6. Compartmental absorption of P3 generated using the GastroPlus® software.

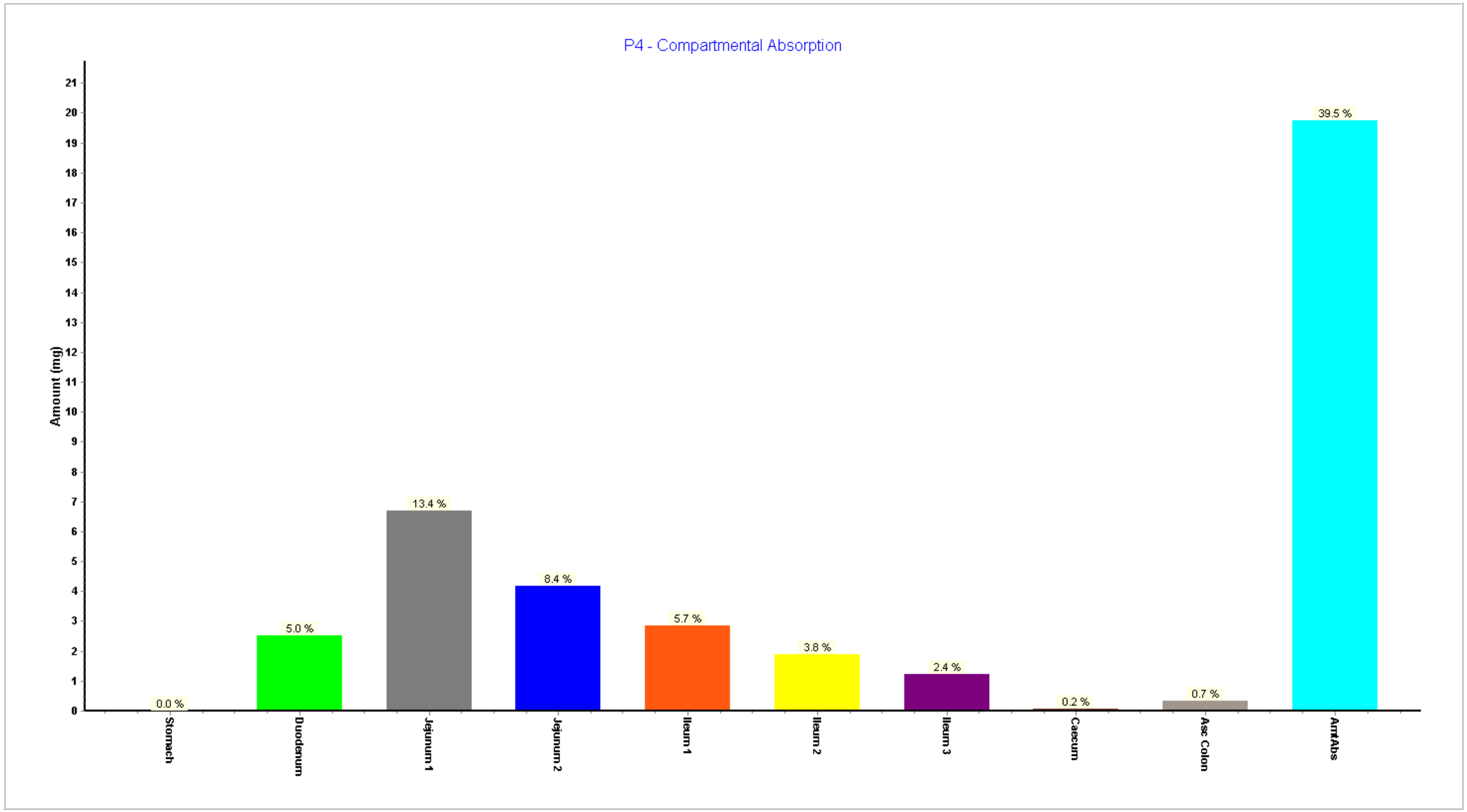

Figure S7. Compartmental absorption of P4 generated using the GastroPlus® software.
